# Untargeted GC-MS and FT-NIR study of the effect of 14 processing methods on the volatile components of *Polygonatum kingianum*

**DOI:** 10.3389/fpls.2023.1140691

**Published:** 2023-05-08

**Authors:** Yulin Xu, Meiquan Yang, Tianmei Yang, Weize Yang, Yuanzhong Wang, Jinyu Zhang

**Affiliations:** ^1^ Medicinal Plants Research Institute, Yunnan Academy of Agricultural Sciences, Kunming, China; ^2^ School of Agriculture, Yunnan University, Kunming, China

**Keywords:** *Polygonatum kingianum*, processing methods, untargeted GC-MS, FT-NIR, volatile components

## Abstract

**Introduction:**

*Polygonatum kingianum* is a traditional medicinal plant, and processing has significantly impacts its quality.

**Methods:**

Therefore, untargeted gas chromatography-mass spectrometry (GC-MS) and Fourier transform-near-infrared spectroscopy (FT-NIR) were used to analyze the 14 processing methods commonly used in the Chinese market.It is dedicated to analyzing the causes of major volatile metabolite changes and identifying signature volatile components for each processing method.

**Results:**

The untargeted GC-MS technique identified a total of 333 metabolites. The relative content accounted for sugars (43%), acids (20%), amino acids (18%), nucleotides (6%), and esters (3%). The multiple steaming and roasting samples contained more sugars, nucleotides, esters and flavonoids but fewer amino acids. The sugars are predominantly monosaccharides or small molecular sugars, mainly due to polysaccharides depolymerization. The heat treatment reduces the amino acid content significantly, and the multiple steaming and roasting methods are not conducive to accumulating amino acids. The multiple steaming and roasting samples showed significant differences, as seen from principal component analysis (PCA) and hierarchical cluster analysis (HCA) based on GC-MS and FT-NIR. The partial least squares discriminant analysis (PLS-DA) based on FT-NIR can achieve 96.43% identification rate for the processed samples.

**Discussion:**

This study can provide some references and options for consumers, producers, and researchers.

## 1 Introduction

Functional foods can have several health benefits for the human body. They can prevent malnutrition and have an interventional effect on metabolic diseases (Ahmed et al., 2022; Fu et al., 2022). As the public pays more and more attention to their health, functional food is becoming increasingly popular (Banwo et al., 2021). Therefore, the quality and safety control of functional foods is crucial. Currently, the functional foods sold in the market are generally processed. Processed functional foods can be more easily transported, stored, and enhance the value of the product (Harasym et al., 2020). However, there are some problems with the processing of the products. For example, the processing process is difficult to unify, and there is a lack of corresponding technical guidance specifications. Accordingly, the quality of the product will vary, improper processing methods lead to the loss of nutrients, and even toxic substances can be produced (Pundir et al., 2019). These may lead to deception and even threaten the health of consumers. Based on the above, it is necessary to study the processing quality of functional foods. In doing so, it can provide consumers with a basis for choice and guide the actual production.

The genus *Polygonatum* is distributed in the Northern Hemisphere, half of which is found in the Himalaya-Hengduan Mountains (Xia et al., 2022). Most of the *Polygonatum* species are considered to have nutritional and medicinal value in China and India (Sharma et al., 2021; Xia et al., 2022). *Polygonatum kingianum* Coll. et Hemsl is a well-known functional food in China and has been used since the Qing Dynasty. It has an edible and medicinal value and makes an important contribution to the economy of China’s mountainous regions. The main chemical components of *Polygonatum* species are polysaccharides, steroidal saponins, triterpenoid saponins, and so on (Zhao et al., 2018). The polysaccharides are the quality evaluation index of *P. kingianum* and have been proven to have the effects of treating diabetes and hypertension (Gu et al., 2020), anti-tumor, anti-oxidation, and anti-aging (Li et al., 2018). The quality of *P. kingianum* is controlled by many factors, such as geographical cultivation area, growing age, processing method, and storage conditions (Xu et al., 2022a). The processing method can directly affect the compositional changes of *P. cyrtonema*, with reactions or decomposition between chemical components (Fan et al., 2020). Variations in these components can affect the sensory quality of *P. kingianum*, leading to important physical changes. *P. kingianum* is often made into dried fruit for snacking, powdered and added to food or drinks as a nutritional supplement, or cooked as a vegetable (Wu et al., 2012). *P. kingianum* is usually sold after processing, as eating it raw can irritate the throat. The common processing methods of *P. kingianum* in the Chinese market include air drying, roasting, steaming, wine steaming, nine times steaming with nine times roasting, etc. (Fan et al., 2020). Yu et al. (2010) analyzed the processed *P. kingianum* using high-performance liquid chromatography (HPLC) and found that the content of specific steroidal saponins was significantly higher than that of the fresh samples. Jin et al. (2021) used HPLC to study the effect of processing on the polysaccharides in *P. kingianum*. The results showed that steaming time, slice thickness, and drying temperature all affected the polysaccharide content. Wang et al. (2022) used chromatography-tandem mass spectrometry (UHPLC-MS/MS) to study the changes in the content of five components during the processing of wine for *P. kingianum* and obtained that the changes in the content of these components were related to the processing time. Fan et al. (2020) used HPLC-MS/MS to analyze *P. cyrtonema* from the processing of steaming and roasting nine times and found that the polysaccharide composition differed greatly between each processing. Generally, the processing research of *P. kingianum* is still in the primary stage, with shallow research levels and single technology. Most of the current studies, which focused on the variation of polysaccharide content, did not perform a comprehensive analysis. Therefore, it is necessary to conduct a comprehensive analysis of 14 kinds of processed *P. kingianum* in the Chinese market. This study may provide an evaluation and control of the processing quality of *P. kingianum*.

Untargeted gas chromatography-mass spectrometry (GC-MS) techniques are commonly used to analyze volatile organic compounds. The good analytical efficiency of gas chromatography and the coupling of mass spectrometry allow the structure of analytes to be determined and initial identification to be performed (Majchrzak et al., 2018). Untargeted GC-MS is widely used in food quality certification, including processing analysis, geographic traceability, adulteration detection, etc. (Farag et al., 2020). This technique has the advantage of high throughput analysis of numerous metabolites. In this study, untargeted GC-MS was used to quantitatively assess the metabolites of *P. kingianum* with different processing methods. However, simultaneously, the technique is costly and takes a long time to analyze. Near-infrared spectroscopy (NIR) has complementary functions with untargeted GC-MS technology. NIR is a prevalent non-destructive analysis technique with the advantage of simple, fast, and comprehensive analysis (Xu et al., 2022a). The NIR spectral data show the absorption intensity of functional groups, which is an overall measurement and more suitable for qualitative evaluation. However, there is no study on the different processing methods of *P. kingianum* by NIR. Therefore, both untargeted GC-MS and Fourier transform-near-infrared spectroscopy (FT-NIR) techniques were chosen in this study to achieve a comprehensive quantitative and qualitative evaluation. The combined use of untargeted GC-MS and FT-NIR can compensate for the disadvantages and build on both advantages. The latitudes of untargeted GC-MS and FT-NIR data were large, thus requiring multivariate statistical analysis. Principal component analysis (PCA) and hierarchical cluster analysis (HCA) are exploratory data analysis methods that allow the low-dimensional representation of high-dimensional data (Borras et al., 2015). In this study, untargeted GC-MS and FT-NIR analyses were performed using PCA and HCA to explore the clustering and distribution among the samples. For further analysis of FT-NIR, the samples were classified using a supervised partial least squares discriminant analysis (PLS-DA) classification model. PLS-DA is often used in multivariate statistical analysis, which is suitable for small samples and can well handle multicollinearity data (Cosme et al., 2021). The PLS-DA model aims to separate samples of different groupings by finding a linear subspace of optimal explanatory variables (Gromski et al., 2015). The PLS-DA model enables the predictive classification of unknown samples by modeling a large number of known sample data. Notably, FT-NIR shows broad and highly overlapping absorption bands, and various types of preprocessing methods are required to improve the interpretation of the spectra.

This study is the first to analyze 14 common processing methods of *P. kingianum* in the Chinese market. Using untargeted GC-MS and FT-NIR technology, combined with multivariate data analysis methods, the qualitative and quantitative evaluation of the quality of different processed *P. kingianum* was realized. This study provides a reference for consumer choice and a theoretical basis for the production of *P. kingianum*.

## 2 Materials and methods

### 2.1 Plant materials

*P. kingianum* samples were collected from Longtan Township, Pu’er City, Yunnan Province. All the collected samples were identified as *P. kingianum* by Professor Jinyu Zhang (Institute of Medicinal Plants, Yunnan Academy of Agricultural Sciences, Kunming, China). The aerial parts and fibrous roots were removed, then washed and prepared for processing. For each processing method, 400 g of fresh *P. kingianum* was used and subsequently divided into 8 replicate groups of 50 g each. After each processing completed, each replicate was dried to brittle at 55°C and ground into powder. 5 g was taken from each of the eight samples to be mixed for subsequent untargeted GC-MS analysis.

### 2.2 Processing experiment methods

**Figure 1:**
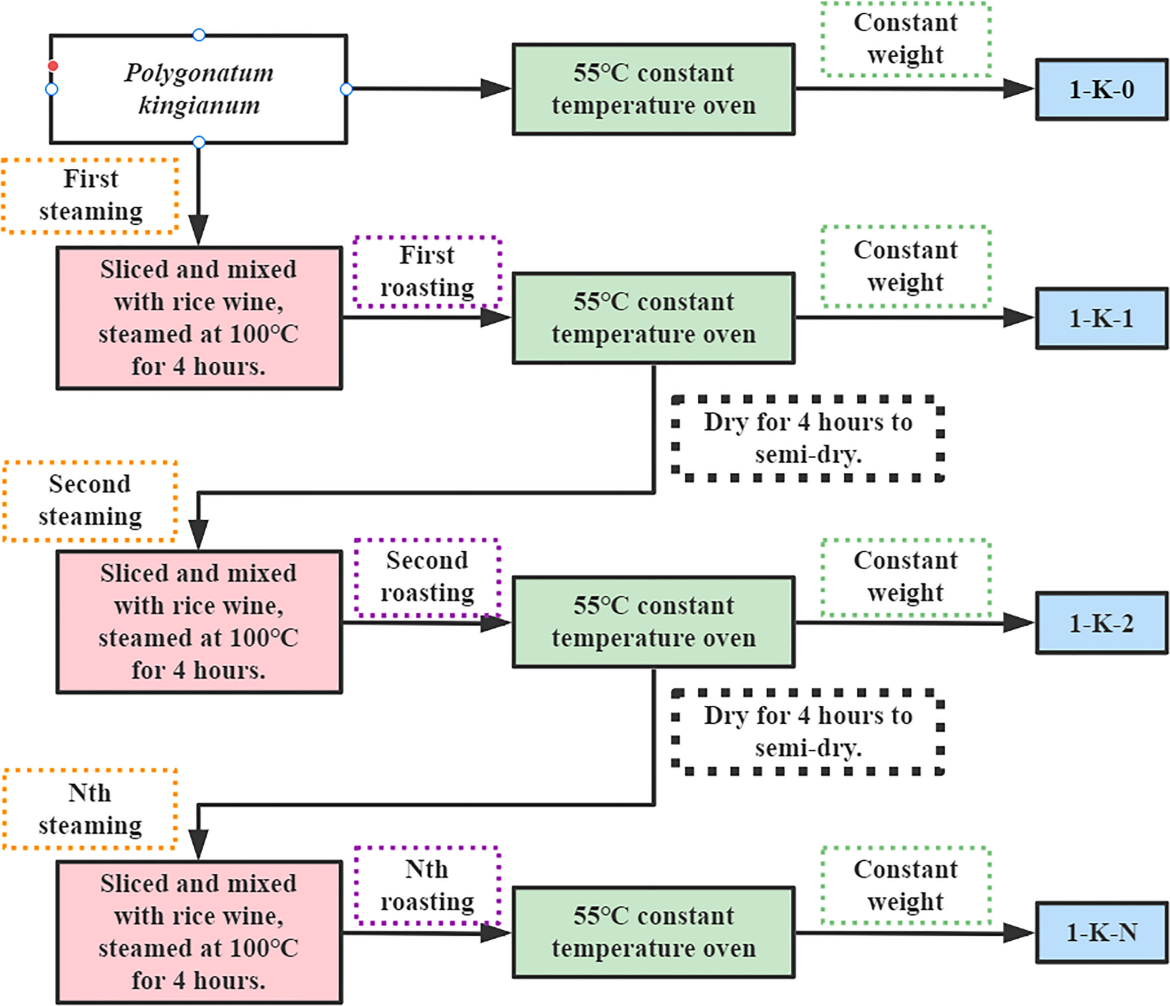
The processing flow of samples (1-k-0 to 1-K-9).

**Figure 2:**
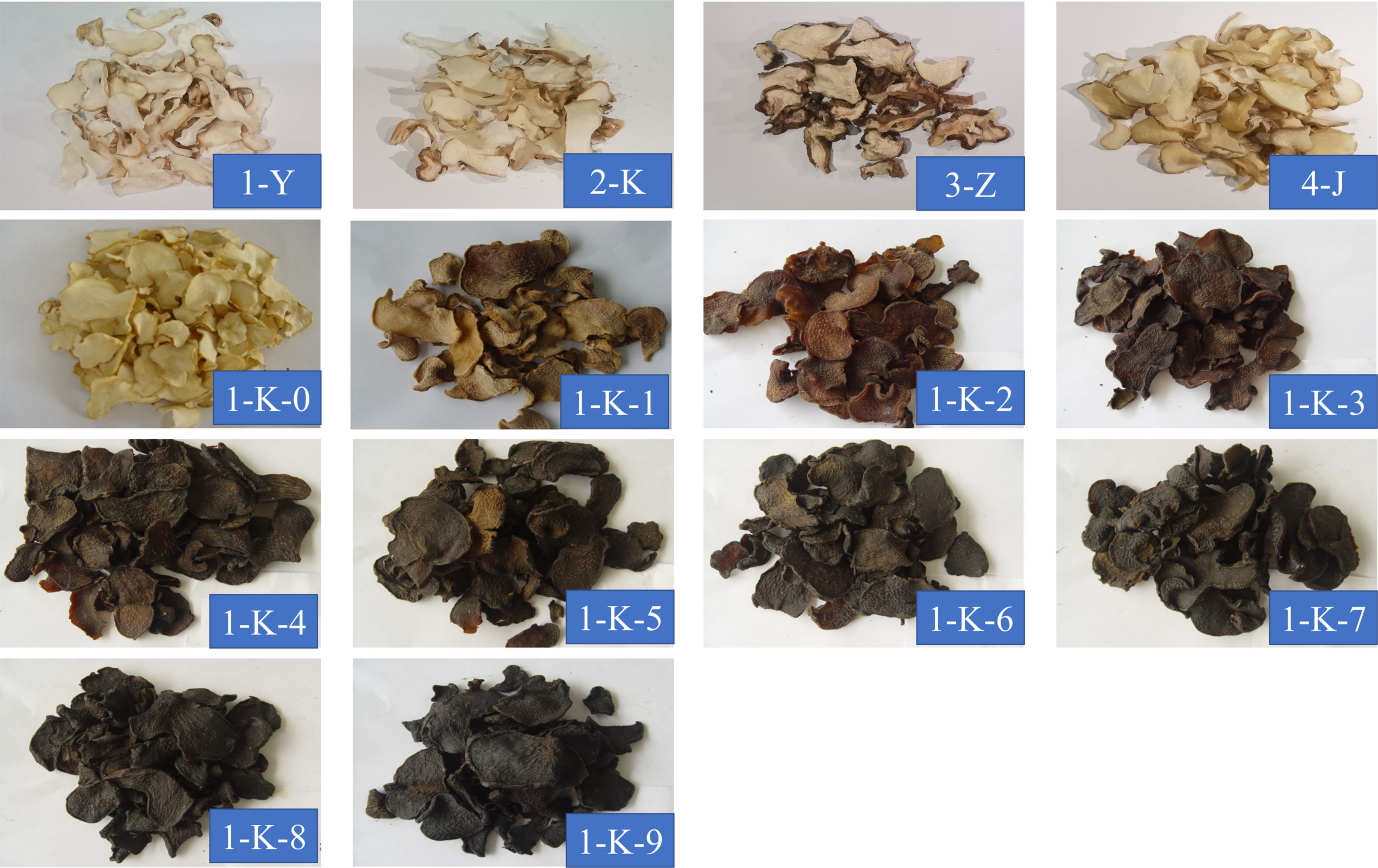
The 14 kinds of processed *Polygonatum kingianum*.

After market research, a total of 14 most common processing methods in the Chinese market were selected. **Dry in the shade (1-Y)**: fresh samples were cut into thin slices and placed in a cool and ventilated place to brittle; **Direct roasting (2-K)**: fresh samples were cut into thin slices and placed in a constant temperature blast drying oven at 55°C to brittle; **Roasting after steaming (3-Z)**: fresh samples were cut into thin slices, placed in a steamer at 100°C for 4 h, and then placed in a constant temperature blast drying oven at 55°C to brittle; **Wine steaming and then roasting (4-J)**: fresh samples were cut into thin slices, mixed with white wine and placed in a steamer at 100°C for 4 h, and then placed in a constant temperature blast drying oven at 55°C to brittle; **Nine times of steaming with roasting (1-K-0 to 1-K-9)**: the slices were mixed with rice wine and placed in a 100°C steamer. After steaming for 4 h, roast them in a constant temperature oven at 55°C for 4 h until semi-dry. This was one steaming and one roasting. Then, repeat the above operation until nine steaming and nine roasting. When one of the processes is finished, they all need to be dried at a constant temperature oven at 55°C to brittle. Among them, 1-K-0 is the original sample. **Figure 1** illustrates the processing flow of the samples (1-K-0 to 1-K-9). **Figure 2** shows 14 kinds of processed *P. kingianum*.

### 2.3 Metabolite extraction

Refer to the experimental method proposed by Fiehn et al. for extracting metabolites (Fiehn and Kind, 2007). The mixed sample of each processing method were accurately weighed 20mg into a 2 mL EP tube, then add 0.5 mL of acetonitrile: isopropanol: water (3:3:2, V/V/V) mixed solution (-20°C) accurately, and add 3-4 2mm zirconium beads; The EP tube was put into a high flux tissue grinder, shocked at 30 Hz for 20 s, allowed to stand for 10 s, cycled eight times, and sonicated in an ice water bath for 5 min; 0.5 mL acetonitrile: isopropanol: water (3:3:2, V/V/V) solution (-20°C) was added again and sonicated in an ice water bath for 5 minutes; After centrifugation at 12,000 rpm for 2 min, 500 μL supernatant solution was taken and added into a new 2 mL EP tube; The EP tube was then put into a vacuum concentrator to concentrate until dry (8-10 h), and the remaining supernatant was placed on -80°C refrigerator for backup; The 80 μL of 20 mg/mL O-Methylhydroxylamine solution was added for re-dissolution, vortex vibration for 30 s, and incubated at 60°C for 60 min; Finally, 100 μL BSTFA-TMCS (99:1) reagent was added and the reaction was carried out at 70°C for 90 min, centrifuged at 14,000 rpm for 3 min, and 90-100 μL of supernatant was added into the detection bottle; Samples were placed in sealed cuvettes to be tested and processed for GC-TOF upper detection within 24 h. The extraction method we use is derivatized to detect as many metabolites with weak volatility in the samples as possible. It is important to note that complex extraction methods may result in the loss of metabolites and affect the true picture of the compounds in the sample.

### 2.4 Untargeted gas chromatography-mass spectrometry conditions

Metabolites were detected using a Pegasus BT gas chromatography time-of-flight mass spectrometer (LECO, Laboratory Equipment Corporation). Gas chromatography was performed on a DB-5MS capillary column (30 m × 250 μm i.d., 0.25 μm film thickness, Agilent J & W Scientific, Folsom, CA, USA) to separate the derivatives at a constant flow of 1 mL/min helium. 1 µL of the sample was injected in the split mode in a 1:10 split ratio by the auto-sampler. The injection temperature was 280°C. The temperature of the transfer line ion source was 320°C and 230°C, respectively. The programs of temperature-rise were followed by an initial temperature of 50°C for 0.5 min, 15 °C/min rates up to 320°C and staying at 320°C for 9 min. Mass spectrometry was performed using a full scan method with a scan rate of 10 spec/s, electron energy of -70 V, and a solvent delay of 3 min.

Additionally, all samples were mixed with the same amount as quality control samples (QC). In the process of getting on the machine, QC value detection is carried out at intervals of 4 samples to judge the system errors, such as instrument error and sample stability during the detection process.

### 2.5 Fourier transform-near-infrared spectroscopy measurement

The 100-mesh fine powder was taken and analyzed by FT-NIR (Thermo Fisher Scientific INC., USA). Place the sample powder in the NIR sample cup for measurement. A total of 32 scans were performed with a resolution of 8 cm^-1^. The scanning range is 10000-4000 cm^-1^. Each sample was measured three times in parallel, and the average spectrum was taken during analysis.

### 2.6 Statistical analysis

#### 2.6.1 Untargeted GC-MS data processing and multivariate analysis

Firstly, the “mzML” format data from the instrument is converted to “abf” format using ABF Converter software for the next step of data analysis (http://www.reifycs.com/AbfConverter/index.html). Subsequently, the transformed data were analyzed using MS-DIAL software (http://prime.psc.riken.jp/Metabolomics_Software/MS-DIAL/index2.html). The analysis process included peak extraction, baseline filtering, correction, peak alignment, and deconvolution. Then, peaks with a signal-to-noise ratio greater than 3 were screened for background noise elimination. The Fiehn gas database was used for the comparative identification of metabolites (https://fiehnlab.ucdavis.edu/projects/fiehnlib). The metabolite identification criteria are based on an overall scoring of the mass spectrometry ion fragments based on their match, retention time, and retention index (All metabolites exemplified in this study had score values greater than 70, with the majority in 90 and above). Metabolites with a mass spectrometry ion matching degree greater than 70% were selected. The retention times of fatty acid methyl esters (FAMEs) in the samples were determined according to the MS-DIAL software, and then the retention indices (RI) of the other metabolites in the samples were calculated using the FAMEs. The tolerance or margin of error for RI is 10,000, i.e., the absolute value of the difference between the theoretical retention index of the metabolite and the actual retention index in the experiment was less than 10,000. Finally, the peak response values for individual ions were calculated from the extract ion chromatography (EIC) plots extracted by the instrument and used as the relative quantitative values for the metabolites. The peak area is normalized for subsequent analysis, and the value is enlarged by 1000 times to reduce the problem of calculation accuracy in the future. It is worth noting that this experiment uses non-targeted detection, that is, the detection of metabolites in the sample without bias. This can be used to compare the relative levels of the same metabolite in different samples. Then, PCA and HCA were established using SIMCA-P+ 14.0 software (Umetrics, Umea, Sweden) to explore the distribution and clustering of the samples.

#### 2.6.2 FT-NIR data processing and multivariate analysis

The original FT-NIR has noise, stray light, unwanted variables, etc., which reduces the accuracy of the analysis (Xu et al., 2022a). Therefore, proper preprocessing can reduce spectral noise and enhance the spectral features of the relevant properties. The multiplicative scatter correction (MSC) and standard normal variate (SNV) preprocessing methods can eliminate the scattering effect caused by uneven sample particle size (Rinnan et al., 2009). The second derivative (SD) can provide higher spectral resolution and reveal more hidden peak information (Rinnan et al., 2009). Therefore, this study employs these three methods and their combination to optimize the data. FT-NIR was visualized by PCA to analyze to conduct the qualitative analysis of different processing methods. Firstly, the 112 samples were divided into 70% training set (84 samples) and 30% test set (28 samples) using the Kennard-stone algorithm. Then, different preprocessed data were used as the input of the model, and the optimization performance of the preprocessing on the data was compared. R^2^ and Q^2^ were used to evaluate the fitness of the model, and root mean square error of estimation (RMSEE), root mean square error of cross validation (RMSECV), and root mean square error of prediction (RMSEP) were used to judge the robustness of the model. The closer the value was to 0, the better the model. The classification performance of the model was reflected by the accuracy of the training set and test set. Additionally, 200 iterations of permutation tests were performed to assess whether the model was overfitting. The PLS-DA model was established using SIMCA-P^+^ 14.0 software (Umetrics, Umea, Sweden).

## 3 Results and discussion

### 3.1 Metabolite analysis of different processed samples

**Figure 3:**
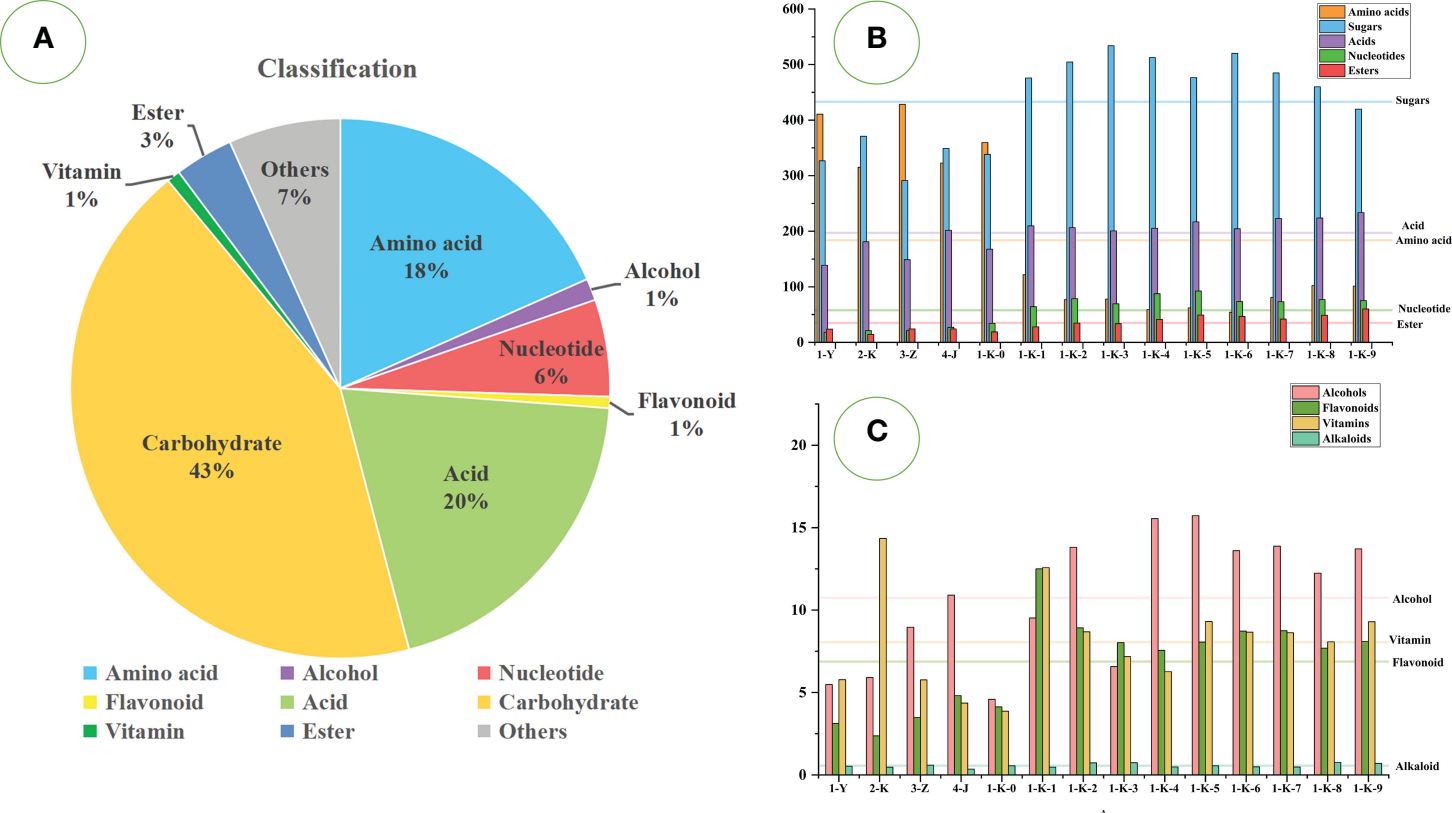
Statistical analysis of GC-MS data of 14 kinds of processed *Polygonatum kingianum*. **(A)** the relative content ratio of various compounds in all samples; **(B)** the relative content of amino acids, sugars, acids, nucleotides, and esters in 14 processed samples; **(C)** the relative content of alcohols, vitamins, flavonoids and alkaloids in 14 processed samples; Each horizontal line in the B and C plots corresponds to the average of the relative contents of the various compounds.

A total of 333 metabolites were identified by the untargeted GC-MS technique. They can be classified into sugars (119 compounds), acids (59 compounds), amino acids (57 compounds), nucleotides (18 compounds), alcohols (19 compounds), esters (13 compounds), flavonoids (5 compounds), alkaloids (4 compounds), vitamins (5 compounds), and others (34 compounds). The relative content accounted of sugars (43%), acids (20%), amino acids (18%), nucleotides (6%), esters (3%), and the rest were relatively low. **Figure 3A** shows the proportions of various compounds. As shown in **Figures 3B, C**, different processing methods have significant effects on various compounds in *P. kingianum*.

#### 3.1.1 Sugars and their derivatives

Sugars are the most important primary metabolite class in *P. kingianum* and are the main energy source required to maintain human life activities. Relatively high sugars are hexose, L-sorbinose, D-(-)-fructose, alpha-D-glucopyranoside, beta-D-fructofuranosyl, L-xylonic acid, D-glucose 1-phosphate, 1F-beta-D-fructosylsucrose, beta-D-glucose, D-tagatose, UDP-glucuronate, etc. The remaining main components are shown in **Table S1**. These are mostly monosaccharides or small molecular sugars. The multiple roasted samples (1-K-1 to 1-K-9) had significantly more sugars than other processing methods. With the increase in roasting times, the sugar content increased upward, reaching a maximum at the third time (1-K-3) and then decreasing. The reason may be that polysaccharides are depolymerized into monosaccharides or small molecular sugars during the steaming and roasting processes (Fan et al., 2020). The sample numbered (3-Z) had the lowest sugar content.

The compounds of hexose, D-(-)-fructose, L-xylonic acid, 3,6-anhydrogalactose, L-(+)-arabinose, and salicin were present in samples (1-Y, 2-K, 3-Z, 4- J, and 1-K-0) with minute amounts. However, the content of these sugars gradually increased with increasing steaming and roasting times and then tended to be stable. Among them, hexose and D-(-)-fructose are common food supplements that contribute significantly to sweetness. UDP-glucuronate, D-raffinose, citrate, beta-D-fructose, and sucrose were relatively abundant in samples (1-Y, 2-K, 3-Z, 4-J, and 1-K-0), but the content gradually decreased with the increase of roasting times. This may be due to the decomposition or reaction with other components during roasting. Studies have confirmed that sucrose is rapidly destroyed after 2 h of roasting, resulting in a sour taste and aroma (Wei et al., 2012). The contents of D-tagatose, inositol, alpha-D-galactose, and turanose were stable in each sample. Among them, inositol belongs to sugar alcohols, which can be produced from sugar and contribute to the sweet taste of *P. kingianum*. It is more thermostable than free sugars (Zayed et al., 2022). Additionally, it also contains some sugar alcohols such as 5-deoxyribitol, D-mannitol, Myo-inositol, lactitol, D-arabitol, D-threitol, butane-1,2,3,4-tetrol, maltitol, etc. Sugar alcohols have high thermal stability and are not prone to Maillard reactions (Wei et al., 2012). Therefore, the content was relatively stable during processing. However, L-sorbinose, Hex-2-ulofuranosyl hexopyranoside, 1-kestose, and beta-maltose showed great differences in each sample, and there was no regularity. Notably, hex-2-ulofuranosyl hexopyranoside accumulated much more in 1-K-7, 1-K-8, and 1-K-9 samples than in other samples. This is because the increase in pyrans/furans content is also a significant feature of the roasting process (Farag et al., 2020). The most abundant pyrans/furans compound is hex-2-ulofuranosylhexopyranoside, in addition to a small amount of alpha-d-xylopyranose, 2-O-methyl-D-mannopyranosa, saligenin-beta-D-glucopyranoside, methyl beta-D- galactoside, etc. Studies have confirmed that amino acids and sugars can generate pyran/furan through the Maillard reaction during roasting (Farag et al., 2015). Six sugars (beta-D-glucose, 1F-beta-D-fructosylsucrose, D-glucose 1-phosphate, 4-O-hexopyranosylhex-2-ulofuranose, melezitose and beta-D-glucosamine) in samples (1-Y, 2-K, 3-Z, 4-J and 1-K-0) were less than the samples (1-K-1 to 1-K-9). With the increase of roasting times, these components showed an upward trend and then gradually decreased. Beta-maltose is degraded to glucose under heat treatment conditions and can undergo the Maillard reaction (Araki et al., 2022). Therefore, the increase and decrease in glucose content may be caused by the degradation of maltose and the Maillard reaction simultaneously.

#### 3.1.2 Acids and their derivatives

Acids have an important influence on the flavor of *P. kingianum* products. A total of 59 acids were detected, but only 19 were abundant, including inorganic acids, organic acids, and fatty acids. The primary acid compounds are phosphoric acid, malic acid, glycolic acid, lactic acid, hexadecanoic acid, octadecanoic acid, etc. The remaining main components are shown in **Table S1**. Acids are lower in samples (1-Y, 3-Z, and 1-K-0), and the most in the 1-K-9. Generally, the processing method has little effect on the acid content of *P. kingianum*.

Phosphoric acid was the only inorganic acid detected in the samples, and it was the most abundant acid species. Phosphoric acid was abundant in all samples with little difference, contributing to the sour taste of *P. kingianum* products (Zayed et al., 2022). Organic acids play an important role in foods’ flavor, color, and aroma (Bouhlali et al., 2020). Many organic acids are often used as sour additives in food, directly affecting the consumer’s taste perception through sourness or acidity. Malic acid is abundant in the roasting processed products of *P. kingianum*. It is a common organic acid with significant antioxidant activity (Nazir et al., 2020). Glycolic acid was less in the samples (1-Y, 2-K, 3-Z, 4-J, and 1-K-0), but the content gradually accumulated with increasing roasting times. Lactic acid, hexadecanoic acid, octadecanoic acid, 5-hydroxymethyl-2-furancarboxylic acid, tartronic acid, and phosphoenolpyruvate acid were not significantly different among the various processed samples. Among them, glycolic acid and lactic acid can directly inhibit the formation of melanin in melanocytes, thereby lightening the color of the skin (Usuki et al., 2003). Hexadecanoic acid and octadecanoic acid are the most common saturated fatty acids in animals and plants. 5-hydroxymethyl-2-furancarboxylic acid is a metabolite produced from 5-hydroxymethyl-2-furfural and can be eliminated renally (Jöbstl et al., 2010). Tartronic acid inhibits the conversion of sugars into fats in the human body (Shi et al., 2020). The samples (1-Y, 2-K, 3-Z, 4-J, and 1-K-0) contained less D-glyceric acid, maleic acid, succinic acid, oxalacetic acid, ethylmalonic acid, 2-hydroxybutanoic acid, and linoleate. However, these components were more abundant in the multiple roasted samples with less significant differences. This result indicates that the use of rice wine for processing is beneficial to the accumulation of these acids, but does not change significantly with the increase in roasting times. It is worth noting that the content of oxalic acid in samples 1-K-8 and 1-K-9 was minimal. 2-Butene-1,4-dicarboxylic acid and 4-hydroxybutanoic acid were more abundant in samples (1-Y, 2-K, 3-Z, 4-J, and 1-K-0), but in the remaining samples rare. Sugars and acids are the two most common compounds in *P. kingianum* products. Its proper proportion balance coupled with solid flavor and aroma is an essential factor influencing consumers’ choice.

#### 3.1.3 Amino acids and their derivatives

Amino acids can act as elements of essential proteins and are also involved in a wide variety of biochemical and physiological processes (Ribarova, 2018). Many studies have shown that the processing and cooking methods of foods have a significant impact on the type and content of amino acids (Motta et al., 2020). A total of 57 amino acids were detected in all samples, including 19 proteinogenic amino acids. The 19 kinds of proteinogenic amino acids include eight kinds of essential amino acids, two kinds of conditionally essential amino acids, and nine kinds of non-essential amino acids. The amino acids with higher content are L-pyroglutamic acid, 5-hydroxy-L-tryptophan, gamma-aminobutyric acid, L-proline, L-tyrosine, L-serine, L-isoleucine, glycine, L-leucine, etc. The remaining main components are shown in **Table S1**. It was clearly observed that most of the amino acids were abundant in the samples (1-Y, 2-K, 3-Z, 4-J, and 1-K-0), but few in the rest of the samples. It is strongly influenced by the processing methods. The present results are similar to those of previous studies, where heat treatment significantly reduced the amino acid content (Chen et al., 2015).

L-Pyroglutamic acid was detected at high levels in the samples and is commonly thought to be associated with browning reactions in food. The production of L-pyroglutamic acid is probably due to the conversion from L-glutamine to L-glutamate first and then to L-pyroglutamic acid during the heating process (Airaudo et al., 1987). The analysis showed that the conversion of L-glutamine and L-glutamate in samples (2-K and 1-K-1 to 1-K-9) was relatively complete. Gamma-aminobutyric acid accumulated more in the samples (1-Y, 2-K, 3-Z, 4-J, and 1-K-0) and none in the rest of the samples. L-Pyroglutamic acid has been studied as a precursor of gamma-aminobutyric acid (Wegener et al., 2017). Gamma-aminobutyric acid has multiple pharmacological activities and is widely used in functional foods (Pandey et al., 2021). The samples (1-K-1, 1-K-7, 1-K-8, and 1-K-9) contained more 5-hydroxy-L-tryptophan. 5-hydroxy-L-tryptophan is considered a popular dietary supplement and is the immediate precursor for the conversion of L-tryptophan to 5-hydroxy-tryptamine (Das et al., 2004). Carnitine and 2-furoic acid were almost absent in the samples (1-Y, 2-K, 3-Z, 4-J, 1-K-0, and 1-K-1), but the contents gradually accumulated with the increase of roasting times. Carnitine is an amino acid derivative, a dietary supplement with a wide range of biological activities, which can be synthesized from L-lysine and L-methionine (Bremer, 1983; Dabrowska and Starek, 2014). Some studies have confirmed that L-lysine is a clear indicator of the heating process of food products and increases with increasing roasting times (Cartus, 2017). However, no such pattern was found in this test. Low levels of three biogenic amines, tyramine, putrescine and cadaverine, were detected in the 1-Y. It is important to note that the presence of biogenic amines in food is closely related to spoilage. The presence of high levels of biogenic amines can affect the quality and safety of food products (Dabadé et al., 2021). Glycyl-L-proline was detected in the samples (1-Y, 2-K, 3-Z, 4-J, and 1-K-0) and was the only cross-linked amino acid detected. The production of such substances may reduce the bioavailability of amino acids (Cartus, 2017). Six odour-defining amino acids, L-glutamate, L-aspartate, L-phenylalanine, L-alanine, glycine, and L-tyrosine were detected in the samples (1-Y, 2-K, 3-Z, 4-J, and 1-K-0). L-Leucine, isoleucine, and L-valine can work together to repair muscles and control blood sugar. In addition to the above-mentioned, other protein-derived amino acids with high content were also detected, such as L-proline, L-serine, L-isoleucine, L-allothreonine, L-threonine, and DL-aspartic acid. Other amino acids include DL-alanine, L-pipecolate, L-ornithine, L-citrulline, DL-homoserine, N-acetylserotonin, and N-acetylornithine were also detected.

#### 3.1.4 Nucleotides and their derivatives

Exogenous nucleotides in food are considered conditionally essential in modern research and play an important role in the life activities of living organisms (Domínguez-Álvarez et al., 2017). A total of 18 nucleotides were detected, of which seven were relatively high in content. Cytosine arabinoside monophosphate was the most abundant nucleotide, with comparable amounts in the samples (1-Y, 2-K, 3-Z, 4-J, and 1-K-0). With the increase of roasting times, the content of cytosine arabinoside monophosphate also increased, reaching the maximum in the 1-K-5 and then decreasing slightly. 5-Methylcytosine was almost absent from the samples (1-Y, 2-K, 3-Z, 4-J, 1-K-0, and 1-K-1). 5-Methyluridine was the most abundant in sample 1-K-1, but the content gradually decreased with increasing roasting times. Uridine was present in tiny amounts in the samples (1-Y, 2-K, 3-Z, and 4-J) but gradually accumulated as the number of roasts increased. Uracil, 1-beta-D-ribofuranosyl- was relatively more abundant in 1-K-2, and 4,5-dihydroorotic acid was relatively more abundant in 2-K. Overall, samples 1-K-5 contained the highest amount of total nucleotides.

#### 3.1.5 Esters and their derivatives

Esters are the aromatic substances found in *P. kingianum*, and a total of 13 were detected. As a whole, esters accumulated progressively with increasing roasting, with the highest levels in 1-K-9. 3-Hydroxypropanoate was the most abundant of the esters, followed by erythronic acid lactone, and they both showed an increasing trend with increasing roasting times. The function of 3-hydroxypropionate is related to a propionate. (https://pubchem.ncbi.nlm.nih.gov/compound/5459847) D-Mannonate was present in small amounts in the samples (2-K, 4-J, 1-K-0, and 1-K-1). D-Mannonate is involved in the metabolism of D-glucuronic acid. (https://ecmdb.ca/compounds/M2MDB000609) The content glyceryl monooleate accumulated in the samples (1-K-4 to 1-K-9) with the increase in roasting times. Glyceryl monooleate is not persistent, bioaccumulative, and toxic, but also has antidiabetes and antioxidant activities, so it is widely used in the food industry (Api et al., 2021; Wei et al., 2021). 3-Hydroxy-3-methylglutarate was relatively the most in sample 1-K-2, and the content gradually decreased after the fifth roasting. 3-Hydroxy-3-methylglutarate has a therapeutic effect on diabetes, but was present in minute amounts in our current study samples (Francesconi et al., 1987).

#### 3.1.6 Miscellaneous

In addition to the above, small amounts of alcohols, flavonoids, vitamins, alkaloids, nitrogenous compounds, etc. were detected. There were 19 alcohols detected. Sphinganine was present in minimal content in samples 1-K-0, but the content accumulated gradually with increasing roasting times. Sphinganine can enhance the adaptability of beneficial microorganisms in organisms (Lee et al., 2021). Glycerol was only present in the samples (1-K-2, and 1-K-4 to 1-K-7) and was reduced the more often they were roasted. 2-Monopalmitin and 2-aminooctadecane-1,3-diol were detected at low levels in all samples. Neohesperidin is the most abundant flavanone glycosides in the samples and has a hypoglycemic effect (Zhang et al., 2012). Three vitamins with high contents were also detected in the samples: Nicotinic acid, pantothenic acid, and ascorbate. Nicotinic acid and pantothenic acid are water-soluble vitamin B compounds involved in life activities and have multiple pharmacological activities (Zeng et al., 2021; Sadok et al., 2022). Ascorbate is commonly used as an antioxidant and acidity regulator in vegetable and fruit products (Jin et al., 2022). Three pyridines were detected: 2-hydroxypyridine, 3-hydroxypyridine, and 4-hydroxypyridine. Pyridines are considered to be frequently produced flavor substances in food processing (Hidalgo et al., 2020). It was found that 2-hydroxypyridine and 3-hydroxypyridine had a significant cumulative effect, accumulating with increasing roasting times. 2-(3,4-Dihydroxyoxolan-2-yl)-2-hydroxyacetaldehyde was rare in the samples (1-Y, 2-K, 3-Z, 4-J, and 1-K-0) and was present in greater amounts in the rest of the samples with no significant differences. 2-Pyrrolidinone was present relatively more in the samples (1-Y, 2-K, and 1-K-0).

#### 3.1.7 Multivariate data analysis of metabolites from differently processed samples

**Figure 4:**
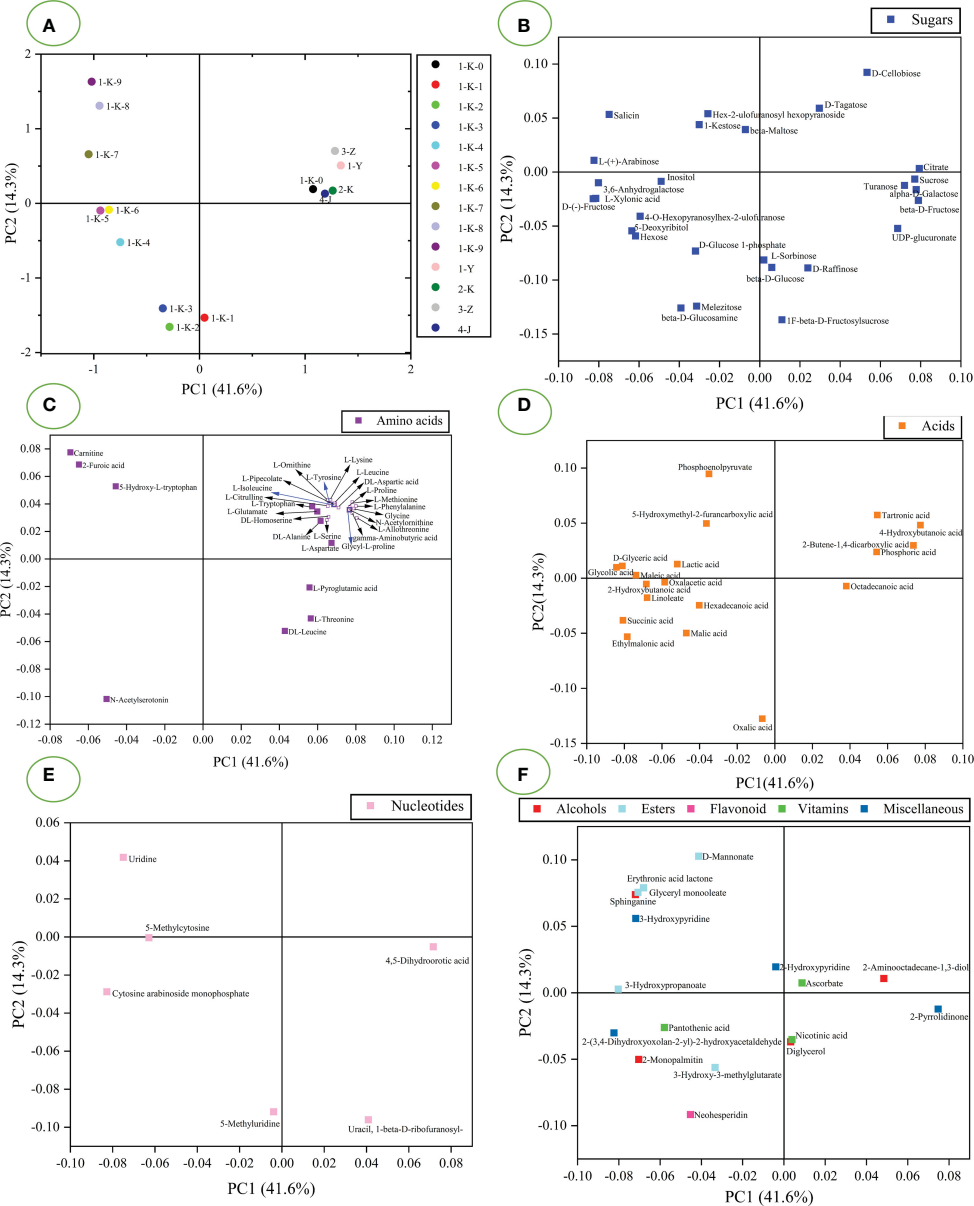
PCA analysis of 14 processed *Polygonatum kingianum* based on GC-MS data. **(A)** the scores plot of PCA; B-F are loading diagrams of PCA, **(B)** sugars; **(C)** acids; **(D)** amino acids; **(E)** nucleotides; **(F)** others.

PCA and HCA modeling were performed from untargeted GC-MS data for all samples. The unsupervised PCA model was used to analyze untargeted GC-MS data to visualize the data and explore the distribution between samples. It can be seen from **Figure S1** that the four quality points are closely clustered, indicating that the experimental method has good stability and fewer instrumental errors. **Figure 4A** shows the score plot for PCA. PC1 accounted for 41.6% of the variance, and PC2 was responsible for 14.3% of the variance. **Figure 4A** shows that the sample has four clusters. Samples 1-Y, 2-K, 3-Z, 4-J, and 1-K-0 were clustered along the right side of PC1. The remaining processed samples are distributed along the negative axis of PC1 (1-K-1 to 1-K-9). The samples (1-K-1 to 1-K-3) have clear clustering, as do samples (1-K-5 and 1-K-6). The samples (1-K-4, 1-K-7, 1-K-8, and 1-K-9) have obvious separation trends. **Figures 4B–F** is the corresponding loading plot of PCA to explain the effect of chemical composition on the sample separation. Loading diagrams were drawn according to the classification of compounds, which are divided into sugars (**Figure 4B**), amino acids (**Figure 4C**), acids (**Figure 4D**), nucleotides (**Figure 4E**), and others (**Figure 4F**). It is observed from **Figure 4B** that citrate, sucrose, turanose, alpha-D-galactose, and beta-D-fructose are the value that contribute significantly to PC1 and can be used as an eigenvalue in the samples (1-Y, 2-K, 3-Z, 4-J, and 1-K-0). The differentiation of sample 1-K-1 from other samples can be attributed to beta-D-glucose, and L-sorbinose. L-(+)-arabinose, D-(-)-fructose, salicin, L-xylonic acid, and 3,6-anhydrogalactose had significantly negative effects on PC1, significantly separating samples (1-K-4 to 1-K-9) and the rest of the samples. These ingredients can serve as markers of the abundance of multiple roasting products. The contribution of amino acids to sample classification is evident in **Figure 4C**. 5-Hydroxy-L-tryptophan, carnitine, N-acetylserotonin, and 2-furoic acid contributed to the negative axis of PC1. These compounds gradually accumulated with the increase of roasting times and could be used as the characteristics of samples (1-K-1 to 1-K-9). As seen in **Figure 4D**, the increase in the number of roasting sessions contributes to the accumulation of acids. 2-Butene-1,4-dicarboxylic acid, tartronic acid, and 4-hydroxybutanoic acid contributed to the positive value of PC1 and were present in minute amounts in samples (1-K-1 to 1-K-9). Oxalacetic acid and phosphoric acid also contributed to the positive PC1 values, but they were significantly greater in the samples (1-K-1 to 1-K-9). Glycolic acid and D-glyceric acid had the most significant effects on the distribution of samples (1-K-1 to 1-K-9). The content of glycolic acid and D-glyceric acid gradually increased with increasing roasting times, which can be used as a marker of roasting times. **Figure 4E** shows that the distribution of nucleotides in the samples varies widely. Uracil, 1-beta-D-ribofuranosyl- and 4,5-dihydroorotic acid phase in samples (1-Y, 2-K, 3-Z, 4-J, 1-K-0, and 1-K-1) more than multiple roasting samples. Cytosine arabinoside monophosphate, 5-methylcytosine, and uridine can all be considered as features in multiple roasting products. 5-Methyluridine may be characteristic of the samples (1-K-1 to 1-K-3). **Figure 4F** shows that esters are more distributed in samples (1-K-1 to 1-K-9). 3-Hydroxypropanoate and 2-(3,4-Dihydroxyoxolan-2-yl)-2-hydroxyacetaldehyde contributed significantly to the distribution of the samples. 2-Pyrrolidinone also contributed significantly to the sample distribution.

**Figure 5:**
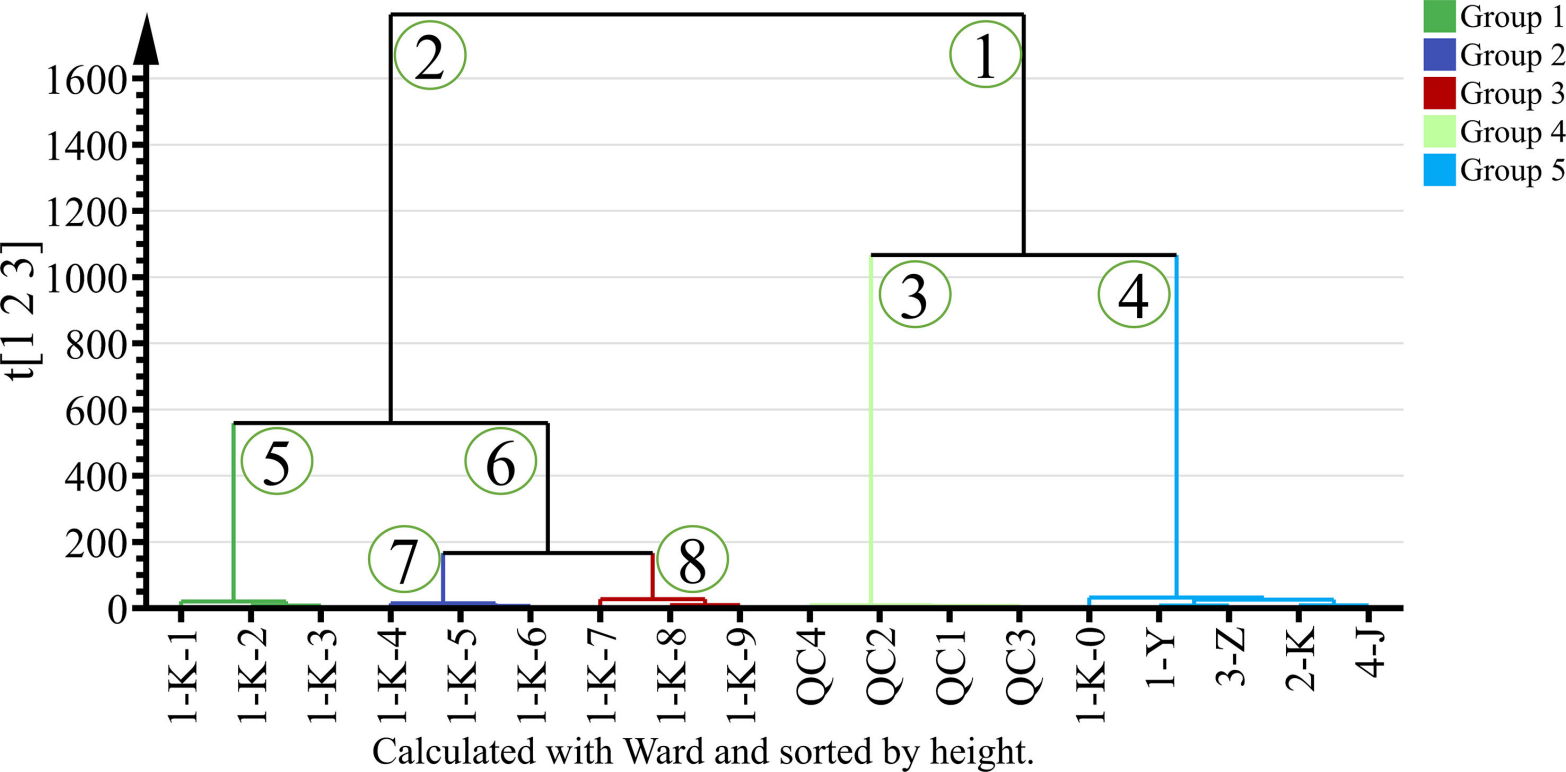
HCA analysis of 14 processed *Polygonatum kingianum* based on GC-MS data.

Different processed *P. kingianum* were classified according to untargeted GC-MS data using HCA. HCA was used to observe the relationship between different samples. The final result is displayed by a dendrogram, which shows the similarities and differences between samples. Computed using Euclidean distance and using Ward’s algorithm to build a dendrogram. Ward’s algorithm provides a classification between samples using the minimum variance method (Taylan et al., 2020). The HCA dendrogram of the sample is presented in **Figure 5**. The samples can be divided into two groups with heterogeneity values up to 1600. Samples with roast multiple times are differentiated in the left arm of the HCA dendrogram. The arm numbered “1” was divided into two clusters, with subclusters numbered “3” and “4”. The arm numbered “3” was the aggregation of quality control samples, which is consistent with the PCA results, indicating the stability of the assay. The samples (1-K-0, 1-Y, 2-K, 3-Z, and 4-J) in the arm numbered “4” were clustered into one group, which is consistent with the PCA results. The arm numbered “2” was divided into two clusters “5” and “6”, and the number “6” was divided into two clusters. In summary, the samples can be divided into 5 clusters according to the HCA of the untargeted GC-MS data.

### 3.2 FT-NIR analysis

#### 3.2.1 FT-NIR spectrum feature interpretation

**Figure 6:**
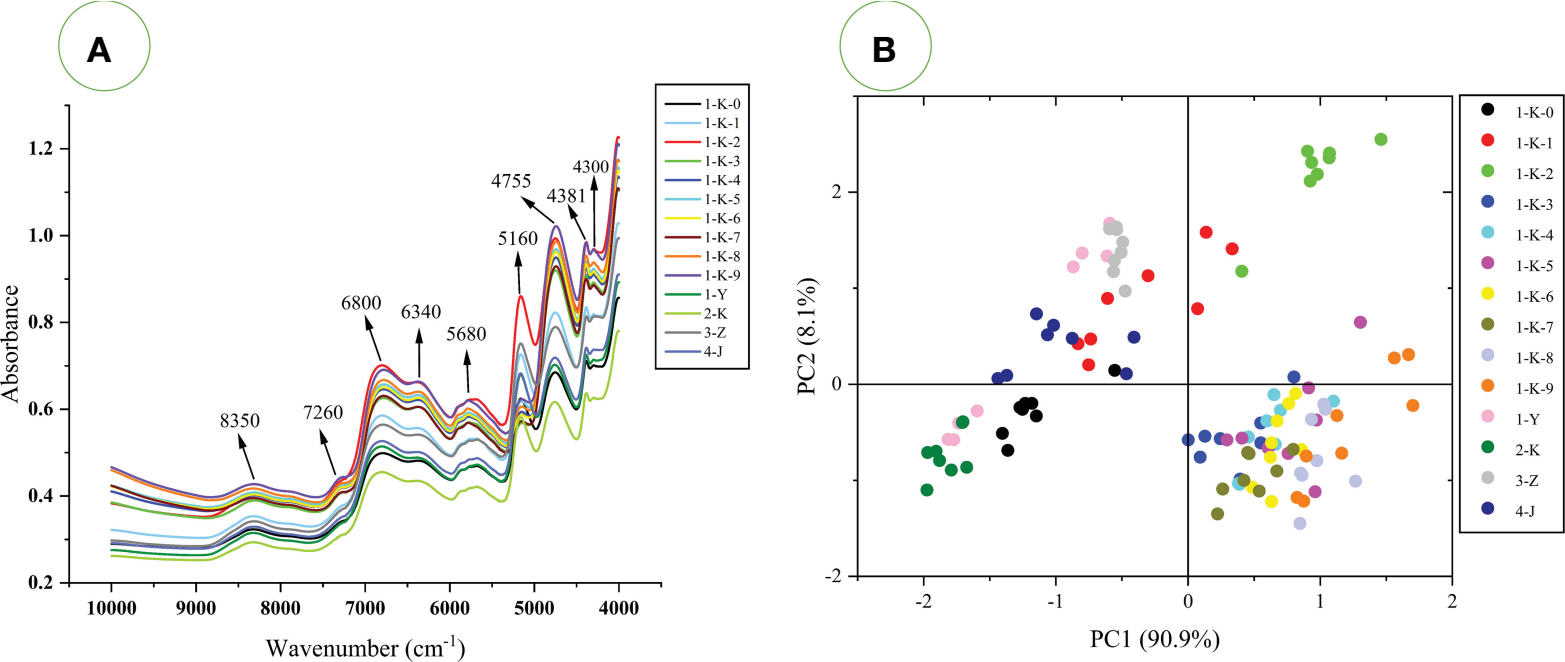
**(A)** The average FT-NIR spectra of 14 processed *Polygonatum kingianum*; **(B)** PCA analysis of 14 processed *Polygonatum kingianum* based on FT-NIR spectra.

The average spectrum for each processing method is shown in **Figure 6A**. **Figure 6A** represents the absorption of C-H, O-H, and N-H chemical bonding vibrations in the samples. High absorbance indicates a high level of compounds containing these chemical bonds in the samples. The spectral peak shapes of each class of processed samples were similar but with significant absorbance differences. This indicates that the content of the chemical constituents of different processed samples has great differences. Nine absorption peaks were observed in the original average spectrum at wavenumbers of 8350 cm^-1^, 7260 cm^-1^, 6800 cm^-1^, 6340 cm^-1^, 5680 cm^-1^, 5160 cm^-1^, 4755 cm^-1^, 4381 cm^-1^, and 4300 cm^-1^. Their formation is associated with the stretching and bending vibrations of the N-H, C-H, and O-H of the hydrogen-containing organic components. According to the literature (López et al., 2017; Ciurczak et al., 2021; Xu et al., 2022b), the FT-NIR was analyzed as follows (1) 4000 cm^-1^ is considered to be caused by the combined vibration of C-H/C-C/C-O-C in polysaccharides; (2) 4386 cm^-1^ to 4252 cm^-1^ can be attributed to the C-H stretching vibration in polysaccharides; (3) 4390 cm^-1^ is the combined vibration of O-H/C-O in glucose; (4) 4750 cm^-1^ corresponds to polysaccharides and carbohydrates, which are generated by C=O-O stretching or O-H deformation; (5) 5168 cm^-1^ and 5089 cm^-1^ are considered as characteristic absorption peaks of polysaccharides. It is produced by the first overtone of O-H stretching; Around wavenumber 5186 cm^-1^ may be the O-H stretching and deformation vibration in sucrose, glucose, fructose, polysaccharides or H_2_O (Zhan et al., 2017). (6) 6000 cm^-1^ to 5400 cm^-1^ is attributed to the first overtone of the C-H stretch (Li et al., 2018). The spectral bands near 5959 cm^-1^ to 5865cm^-1^ are assigned as the first overtone of C-H stretching in ketones; (7) 6300 cm^-1^ to 5400 cm^-1^ attributed to the first overtone of C-H stretching (8) 6798 cm^-1^ to 5025 cm^-1^ and 4878 cm^-1^ to 4831 cm^-1^ are generally believed to originate from amides or proteins. The first overtone of N-H stretching (Lima et al., 2022); 6800 cm^-1^ can also be attributed to the first overtone of O-H stretching vibration in H_2_O; (9) 7000 cm^-1^ to 6700 cm^-1^ is the first overtone of C-H group (Chen et al., 2018); 7100 cm^-1^ to 6000 cm^-1^ is the first overtone of the O-H stretch or N-H stretch (Amirvaresi et al., 2021). (10) 7200 cm^-1^ to 6900 cm^-1^ may be due to the first O-H overtone in the polysaccharide; (11) The band near 8354 cm^-1^ is considered as the second overtone of the C-H stretching vibration of methyl or methylene in aliphatic hydrocarbons (Li et al., 2018).

It is worth noting that around 5263 cm^-1^ to 5235 cm^-1^ is the C=O and C=OOH band of carboxylic acids, and around 5128 cm^-1^ to 5102 cm^-1^ is the C=O absorption band of acids and esters (Ciurczak et al., 2021). The sample was rich in acid but showed no characteristic peaks on FT-NIR. This may be because the absorption peaks of the sugars cover the acids, as the sugars also have absorption in this wavenumber region and the sample contains more sugars than acids. Amino acid content varied widely in the untargeted GC-MS data but showed no significant characteristic peaks in the FT-NIR spectra. After reviewing the literature (Cozzolino et al., 2010; Ciurczak et al., 2021; Wang et al., 2021), the absorption of amino acids is at 8333 cm^-1^ (-CH=CH second overtone), 6757 cm^-1^ (first overtone of C-H), 4673 cm^-1^ (C-H vibrations), 4344 cm^-1^ and 4259 cm^-1^ (C-H combination tones), 4587 cm^-1^ to 4566 cm^-1^ (N-H vibrations). Against **Figures 2**, **5**, no higher absorbance was observed for the samples (1-Y,2-K,3-Z, 4-J, and 1-K-0) in any of these bands, the absorption peaks of amino acids are masked by more sugars. In the subsequent analysis, various preprocessing methods were used to reveal hidden peaks. The absorbance values of samples (1-K-2, 3-Z, 1-Y, 1-K-1, and 4-J) at 5160 cm^-1^ are relatively high, which is most likely due to the content of polysaccharides (Ciurczak et al., 2021; Xu et al., 2022b). This is because the polysaccharides are broken down into small molecular sugars or monosaccharides during the multiple steaming and roasting process (Yao et al., 2022). The 4750 cm^-1^ can be attributed not only to polysaccharides, but also to carbohydrates (Ciurczak et al., 2021). Considering polysaccharides and small molecular sugars together, this may account for the higher absorbance of the samples (1-K-9 and 1-K-2). This may also be why it differs from that at 5160 cm^-1^. Samples 1-K-2 and 1-K-9 had higher absorbance at 6800cm-1, and the multiple steaming and roasting samples were also significantly higher than the samples (1-Y, 1-K-0, 2-K, and 4-J). Therefore, the 6800 cm^-1^ peak in **Figure 5** may be due to the increase in bound water in the sample as the number of steaming and roasting cycles increases (Liu et al., 2021). According to our practical experience, the samples with more steaming and roasting times are easier to regain moisture. The high absorbance of the 1-K-2 sample may be due to the high polysaccharide content. Although the peak at 6800 cm^-1^ can also be attributed to the N-H bond of amide or protein. There is no inclination to interpret the peak at 6800 cm^-1^ as an absorption of amide or protein. This is because absorption is present at 6798 cm^-1^ to 5025 cm^-1^ and 4878 cm^-1^ to 4831 cm^-1^, but no characteristic peak is shown at any other wavenumbers (Ciurczak et al., 2021).

In general, the absorbance intensity of the average spectrum of the multiple roasting is generally higher than that of the rest of the processing methods. According to GC-MS data and literature analysis, the higher absorbance of multiple roasting samples may be due to the higher content of sugars and nucleotides. The absorbance intensities of 1-K-9 and 1-K-2 in the samples were relatively higher, and the absorbance of 2-K was the lowest. The low absorbance values for sample 2-K may be attributed to the low content of both small molecule sugars and polysaccharides. The 2-K samples contained more amino acids compared with the samples (1-K-1 to 1-K-9), but the lower percentage of amino acids could not compete with the sugars. Sample 2-K also had lower absorbance values compared to (1-Y, 3-Z, 4-J, and 1-K-0). The reason for this may be that the polysaccharide content is also lower, especially at 5160 cm^-1^. It is worth noting that the absorbance value of a particular wavenumber cannot be attributed entirely to one substance, but is probably to be the combination of many substances. The FT-NIR spectra of each sample are pretty different, but it is still difficult to visually distinguish each sample. Therefore, the supervised PLS-DA models were used for more in-depth analysis.

#### 3.2.2 Principal component analysis based on FT-NIR

An unsupervised PCA model was built using raw FT-NIR spectra to explore clustering between samples. The PCA score plot is shown in **Figure 6B**. Three distinct clustering trends were observed using PC1 and PC2, explaining 99% of the model variation. PC1 contributes 90.9% of the variance, mainly for distinguishing the multiple roasted samples (1-K-2 to 1-K-9) and the rest of the processed samples (1-Y, 2-K, 3-Z, 4-J, 1-K-0, and 1-K-1). The multiple roasted samples were distributed in the positive direction of PC1, and the remaining samples were distributed in the negative direction of PC1. PC2 is responsible for 8.1% of the variance and mainly contributes to the separation of the samples (1-K-2). PCA based on FT-NIR or GC-MS gave similar results, and it is speculated that the clustering is more related to the amino acids and sugars in the samples. The most significant difference between the two results is that the sample (1-K-2) was independently distinguished in FT-NIR. This may be due to the correlation of the highest absorption peak around the wavenumber 5160 cm^-1^. Although three distinct clusters were obtained using PCA, the classification among the groups was not sufficiently clear, so supervised PLS-DA models were used for the next step of the analysis.

#### 3.2.3 Partial least squares discriminant analysis based on FT-NIR

Supervised PLS-DA models were built using different preprocessed FT-NIR data. To ensure the reliability of the model, 7-fold cross validation was used to build the model. Moreover, the most suitable number of potential variables (LVs) were selected by the lowest RMSECV and the highest Q^2^. All the model parameters were recorded in **Table 1**. The closer the values of RMSEP, RMSEE, and RMSECV are to zero, the stronger the robustness of the PLS-DA model. From all model results, the model built from the original spectra gave the best classification results. The accuracy of the model’s training set and test set were 95.24% and 92.86%, respectively. RMSEP, RMSEE, and RMSECV were 0.1607, 0.1636, and 0.1908, respectively. From the confusion matrix in **Figure S2**, the identification of samples (1-K-7) is the most challenging and is mistakenly identified as 1-K-6, 1-K-8, and 1-K-9. The model built by MSC preprocessing has the following best recognition effect. The training and test sets achieved correct recognition rates of 90.48% and 96.43%, respectively. RMSEP, RMSEE, and RMSECV were 0.1523, 0.1624, and 0.1929. It is worth noting that the test set of the model has a higher correct rate than the training set. The 200 iterations of permutation tests (**Figure S3**) of the model did not reveal any risk of overfitting, so the reason for this is probably the small sample size. As observed from the confusion matrix plot in **Figure S2**, the identification of samples 1-K-6 and 1-K-7 had identification barriers after preprocessing with MSC. In general, it appears from **Table 1** that the preprocessing did not improve the classification results and was not as effective as the original spectral modeling. The reason for this may be that the spectral preprocessing shows more spectral information but does not extract its feature information, resulting in more redundant and invalid information. The Q^2^ and R^2^ of all models were relatively low, indicating the low robustness and reliability of the models. Therefore, feature selection or increasing the sample size of the spectral data is likely to improve the performance of the model. In summary, the PLS-DA model can identify and differentiate between different processed *P. kingianum* samples to some extent.

**Table 1:** Parameters of the PLS-DA model based on FT-NIR data.

	R^2^	Q^2^	LVs	RMSEP	RMSEE	RMSECV	Train Acc (%)	Test Acc (%)
Original	0.655	0.433	17	0.1607	0.1636	0.1908	95.24	92.86
MSC	0.601	0.404	15	0.1523	0.1624	0.1929	90.48	96.43
SNV	0.594	0.389	15	0.1616	0.1770	0.1974	88.10	89.29
SD	0.635	0.415	11	0.1820	0.1637	0.1995	92.86	71.43
MSC+SD	0.687	0.426	12	0.1673	0.1300	0.2026	98.81	82.14
SNV+SD	0.688	0.428	12	0.1665	0.1518	0.1955	92.86	85.71

R^2^=Coefficient of determination; Q^2^ represents the prediction ability of PLS-DA model.; LVs, Number of potential variables; RMSEP, Root mean square error of prediction; RMSEE, Root mean square error of estimation; RMSECV, Root mean square error of cross validation; Train Acc, Classification accuracy of train sets; Test Acc, Classification accuracy of test sets.

### 3.3 Discussion

The results of this study show that different processing methods significantly affect the chemical composition of *P. kingianum*. The multiple roasted samples have more sugars, nucleotides, esters, and flavonoids, but fewer amino acids. Heat treatment significantly reduces the amino acid content, which is similar to the results of this study (Chen et al., 2015). Acids and alkaloids did not vary much between the various types of processed samples, but there was a little pattern in the distribution of alcohols and vitamins. These chemical components are closely related to the flavor and nutritional value of *P. kingianum*. In terms of sugars, acids, nucleotides, and esters, samples 1-K-3 to 1-K-6 are more nutritious. If the amino acids are chosen, then 1-Y and 3-Z are better. However, it is worth noting that three low levels of biogenic amines were detected in 1-Y. The samples (1-Y, 2-K, 3-Z, 4-J, and 1-K-0) contain more polysaccharides and amino acids and are suitable for use as nutritional supplements. With the increase of steaming and roasting times, they are more suitable for consumption as dried fruit, as the Maillard reaction contributes to the formation of color and flavor. **Figure 1** shows that the color of the samples (1-Y, 2-K, 3-Z, 4-J, and 1-K-0) is close to the original beige of *P. kingianum*. The color of the samples (1-K-1 to 1-K-9) gradually darkens to black with increasing steaming and roasting times. Fan et al. (2020) used HPLC-MS/MS to study polysaccharide changes in *P. cyrtonema* processed by steaming and roasting nine times. The results concluded that the polysaccharides in the processed *P. cyrtonema* samples were mainly composed of galactose, mannose, and glucose. The composition of polysaccharides during processing is very different, and the changes are not parallel. However, hexose, L-sorbinose, D-(-)-fructose, and hex-2-ulofuranosyl hexopyranoside were the most abundant sugars in the study here. The reasons for this may be differences in species, processing, detection, and extraction methods. This study is the first time to use untargeted GC-MS and FT-NIR to explore the effect of processing methods on the chemical composition of *P. kingianum*. However, a limitation of this study is that the experimental parameters of the processing method used may not be optimal. The later research can focus on parameter adjustment and optimize the processing method, such as the time of steaming, the temperature of roasting, and the thickness of the slices. It is also worth noting that the quality of processed *P. kingianum* is evaluated on a multi-factorial basis. As a food product, consumers care more about taste and texture; as a functional product, the more potent chemical composition is more important. It is indisputable that many non-volatile compounds have nutritional value or biological activity. Therefore, the next research may consider using LC-MS for detection.

The FT-NIR spectra show the same trends and different absorbances for the different processed samples. Thus, differences in the chemical composition of different processed samples are indicated. Similar distribution results were obtained by PCA based on GC-MS and FT-NIR. The results confirmed the validity and accuracy of FT-NIR in describing the chemical composition of different processed samples. The PLS-DA model based on FT-NIR achieved 71.43%-96.43% identification accuracy for samples treated with different processing methods. The preprocessed FT-NIR did not improve the performance of the models, and all models had lower R^2^ and Q^2^. This may be because too much redundant information hides information that is powerful for classification. Variable selection or feature extraction might be the solution. For better results in later studies, sample sizes can also be increased, or other analytical instruments can be considered for fusion analysis. After increasing the sample size, the correlation analysis between FT-NIR and GC-MS data can be considered to further confirm the analytical capability of FT-NIR. FT-NIR is the key to enable rapid analysis, and many studies have demonstrated its feasibility in implementing predictive components. In one study, NIR was used to predict soluble solids content in blueberries (Bai et al., 2022).

In conclusion, this study is the first to analyze 14 processed *P. kingianum* using untargeted GC-MS and FT-NIR. It can provide some references and options for consumers, producers, and researchers.

## 4 Conclusions

In this study, we used untargeted GC-MS and FT-NIR to qualitatively and quantitatively evaluate different processed *P. kingianum*. The results showed that the chemical composition of different processed *P. kingianum* differed greatly. Sugars, amino acids and acids are the more abundant components of *P. kingianum*. The sugars were mainly monosaccharides or small molecular sugars and were more abundant in the samples roasted several times. This may be the polysaccharides were broken down during the steaming and roasting process. The samples with multiple roasts contained more nucleotides, esters, and flavonoids. The heat treatment significantly reduces the amino acid content. The acids and alkaloids in different processed samples did not change much. Changes in compounds can be attributed to accumulation, chemical reactions or transformations, presumably closely related to temperature. Overall, the samples (1-Y, 2-K, 3-Z, 4-J, and 1-K-0) contained more polysaccharides and amino acids and were suitable for use as nutritional supplements. With increasing steaming and roasting times, they are more suitable for consumption as dried fruits, as the Maillard reaction promotes the formation of color and flavor. Both PCA and HCA based on untargeted GC-MS data aggregate multiple roasted samples into a group. The loading plot of PCA shows how the different compounds contribute to the clustering of the sample. The FT-NIR spectra of each processed sample had nine absorption peaks with similar peak shapes but significant absorbance differences. The PCA based on FT-NIR observed three clustering trends in the samples that could be attributed to the distribution of sugars and amino acids. Supervised PLS-DA models were established based on FT-NIR with different preprocessing, and the recognition rate could reach 71.43-96.43%. In summary, this study achieved effective qualitative and quantitative evaluation of the different processed *P. kingianum* using untargeted GC-MS and FT-NIR. The results of the study can provide some references and options for consumers, producers and researchers and can also contribute to the industrial development of *P. kingianum*.

## Data availability statement

The original contributions presented in the study are included in the article/**Supplementary Material**. Further inquiries can be directed to the corresponding authors.

## Author contributions

YX: experiment, data analysis, modeling, and writing-original draft. MY: sample resource collection and processing. TY and WY: collected literature and optimization of language; YW and JZ: proofreading the study, project administration, and funding acquisition. All authors contributed to the article and approved the submitted version.
