# Supplementary material for: Untargeted GC-MS and FT-NIR study of the effect of 14 processing methods on the volatile components of Polygonatum kingianum
Source: Front Plant Sci. 2023 May 8;14:1140691. doi: 10.3389/fpls.2023.1140691 (PMC10200983; doi:10.3389/fpls.2023.1140691)
Supplement: Supplementary file 1 [file DataSheet_1.docx]

Supplementary Material


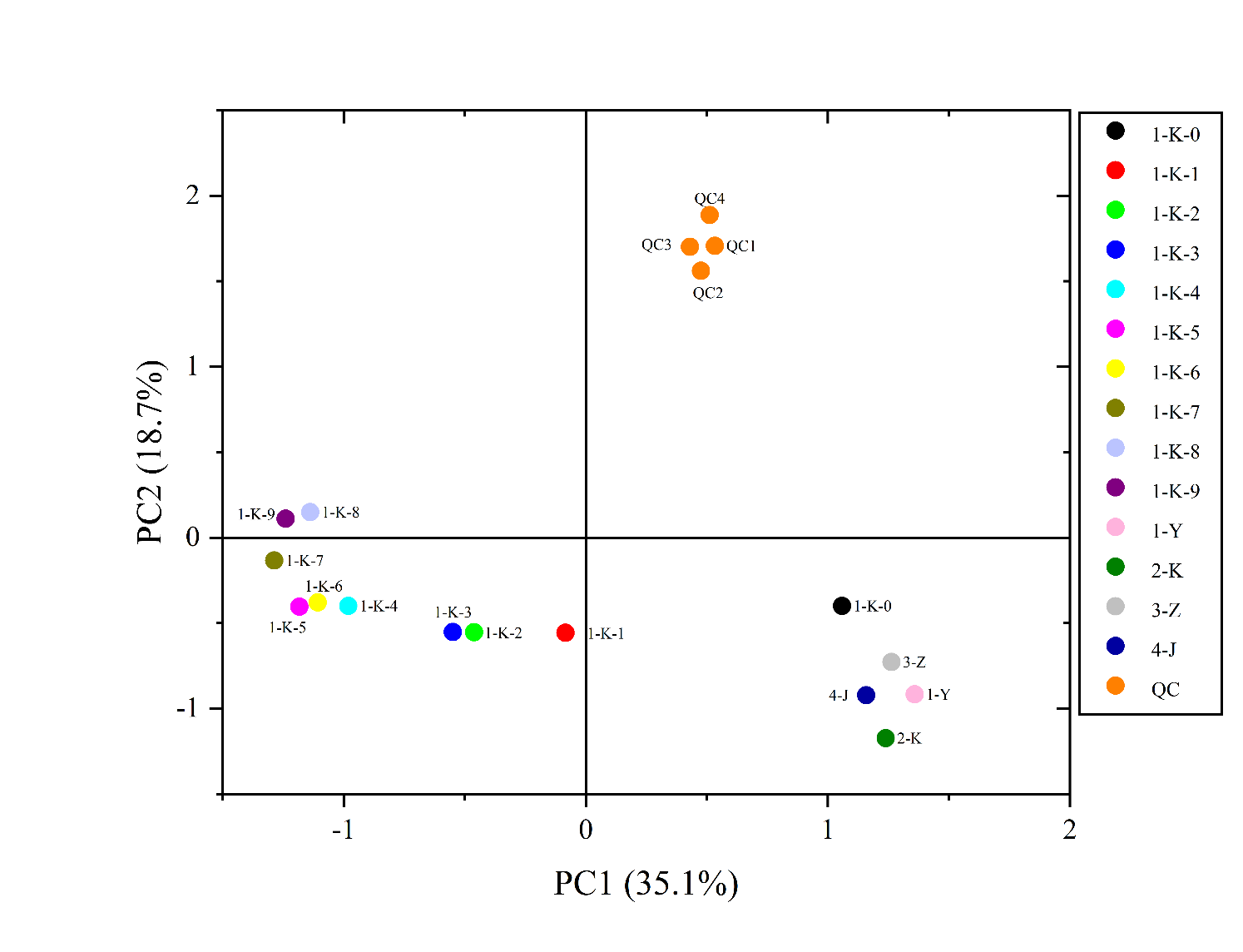


**Figure S1.** PCA analysis of quality control points.


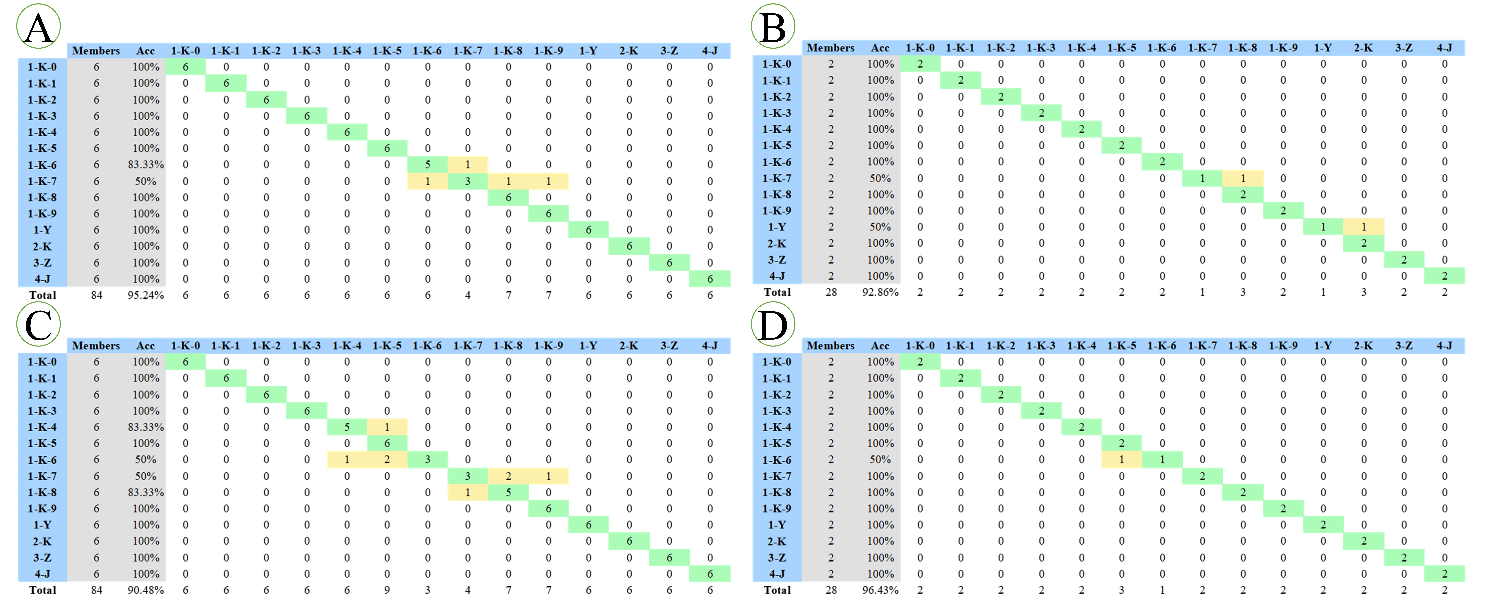


**Figure S2.** Confusion matrix graph with the best performance in PLS-DA model (Original and MSC). A (training set with original), B (testing set with original), C (training set with MSC), and D (testing set with MSC)


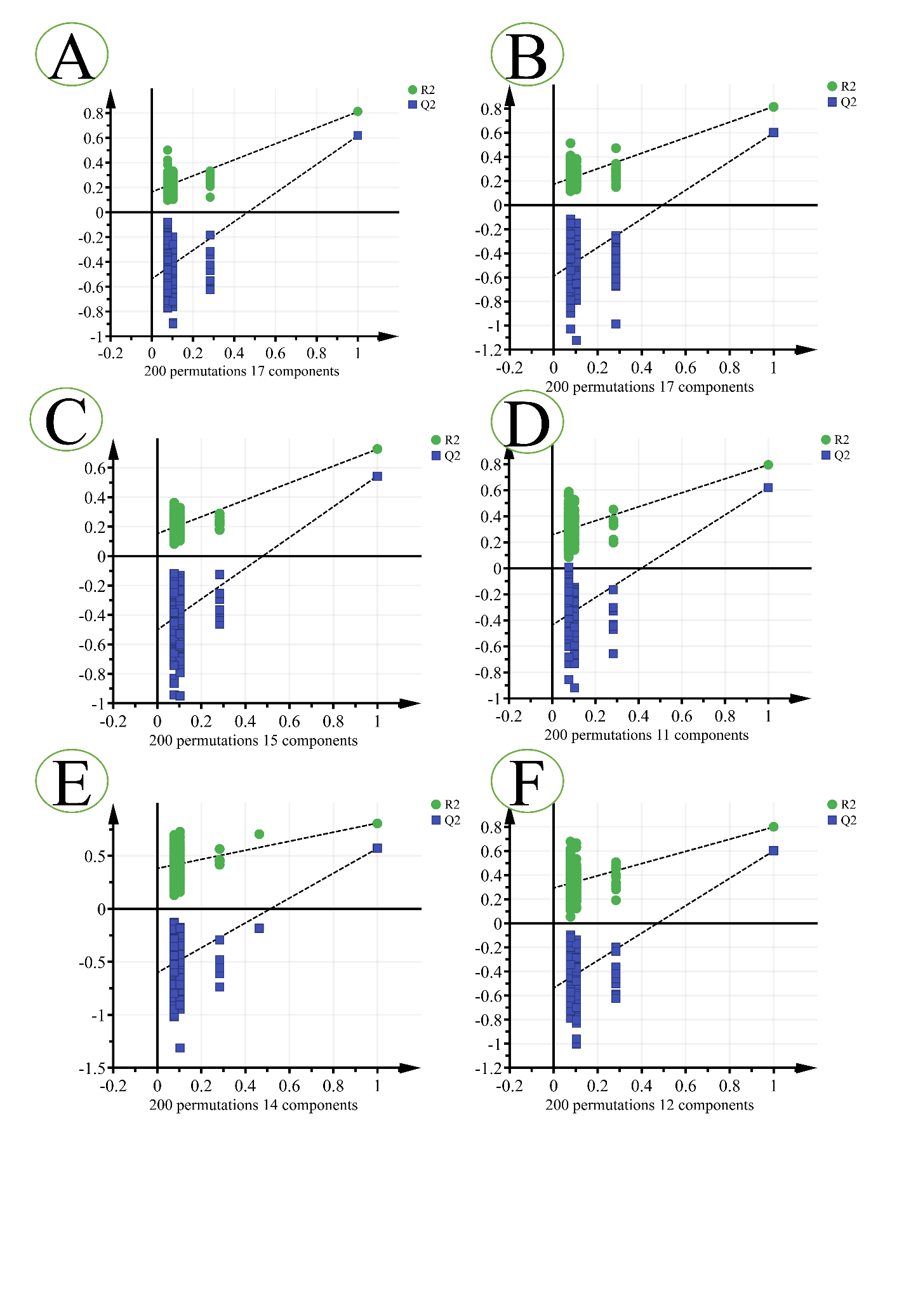


**Figure S3.** 200 permutation iteration tests for PLS-DA models, Original (A), MSC (B), SNV (C), SD (D), MSC+SD (E), SNV+SD (F).

Table S1 The component with the higher relative content of each compound with an average value greater than or equal to 2.

| No | Compound | RT | RI | 1-Y | 2-K | 3-Z | 4-J | 1-K-0 | 1-K-1 | 1-K-2 | 1-K-3 | 1-K-4 | 1-K-5 | 1-K-6 | 1-K-7 | 1-K-8 | 1-K-9 | SUM | AVG | VAR |
| --- | --- | --- | --- | --- | --- | --- | --- | --- | --- | --- | --- | --- | --- | --- | --- | --- | --- | --- | --- | --- |
| Sugars and their derivatives | | | | | | | | | | | | | | | | | | | | |
| 1 | Hexose | 11.144 | 676644.06 | 3.05 | 2.73 | 6.19 | 2.34 | 14.50 | 15.26 | 53.89 | 58.11 | 63.93 | 62.54 | 64.43 | 49.66 | 41.25 | 0.29 | 438.16 | 31.30 | 722.16 |
| 2 | L-Sorbinose | 10.68 | 638890.88 | 16.65 | 20.74 | 18.39 | 25.30 | 21.21 | 24.26 | 34.97 | 32.90 | 18.44 | 4.71 | 33.61 | 26.35 | 21.76 | 6.43 | 305.72 | 21.84 | 81.08 |
| 3 | D-(-)-Fructose | 10.771 | 646349.88 | 4.78 | 5.86 | 4.80 | 5.87 | 5.60 | 29.24 | 27.08 | 27.60 | 32.27 | 34.01 | 30.46 | 31.79 | 31.25 | 32.25 | 302.85 | 21.63 | 161.36 |
| 4 | Hex-2-ulofuranosyl hexopyranoside | 14.091 | 914265.31 | 17.28 | 16.46 | 9.74 | 16.87 | 21.28 | 44.42 | 11.12 | 10.34 | 9.31 | 8.94 | 12.22 | 38.05 | 40.49 | 44.62 | 301.13 | 21.51 | 194.01 |
| 5 | L-Xylonic acid | 10.305 | 608559.94 | 0.86 | 0.75 | 3.12 | 0.60 | 11.59 | 21.85 | 35.24 | 27.34 | 33.83 | 33.35 | 32.89 | 31.42 | 36.12 | 30.09 | 299.05 | 21.36 | 212.36 |
| 6 | UDP-glucuronate | 10.012 | 584968.5 | 25.57 | 39.04 | 17.53 | 25.86 | 21.15 | 24.02 | 24.12 | 20.94 | 5.93 | 7.84 | 5.63 | 4.87 | 3.66 | 7.70 | 233.85 | 16.70 | 116.65 |
| 7 | D-Tagatose | 10.618 | 634002.88 | 15.96 | 20.22 | 13.60 | 16.22 | 12.88 | 12.13 | 7.50 | 8.63 | 10.61 | 16.44 | 14.83 | 17.41 | 10.08 | 12.45 | 188.96 | 13.50 | 12.88 |
| 8 | beta-D-Glucose | 10.879 | 655073.19 | 10.66 | 9.72 | 9.99 | 16.14 | 9.08 | 19.92 | 10.13 | 22.30 | 24.96 | 2.41 | 22.11 | 2.98 | 3.92 | 4.73 | 169.05 | 12.08 | 59.28 |
| 9 | 1F-beta-D-Fructosylsucrose | 18.639 | 1122345.75 | 14.56 | 11.47 | 5.81 | 11.08 | 9.56 | 20.32 | 21.70 | 20.59 | 12.87 | 14.17 | 12.31 | 4.56 | 3.10 | 0.14 | 162.25 | 11.59 | 43.66 |
| 10 | D-Glucose 1-phosphate | 10.056 | 589204.56 | 3.54 | 3.69 | 3.52 | 4.31 | 21.15 | 3.69 | 24.57 | 20.94 | 18.18 | 13.79 | 16.09 | 9.58 | 7.43 | 7.51 | 157.98 | 11.28 | 58.46 |
| 11 | Inositol | 11.754 | 726622.31 | 11.85 | 12.60 | 7.11 | 9.80 | 8.81 | 11.65 | 11.75 | 11.19 | 11.22 | 12.75 | 11.77 | 13.08 | 11.34 | 12.45 | 157.38 | 11.24 | 2.70 |
| 12 | D-Raffinose | 18.584 | 1120809.75 | 15.19 | 8.39 | 12.56 | 7.50 | 6.65 | 12.13 | 10.58 | 14.27 | 10.87 | 11.53 | 13.30 | 4.62 | 3.09 | 2.38 | 133.05 | 9.50 | 17.08 |
| 13 | 3,6-Anhydrogalactose | 10.009 | 585217.88 | 0.81 | 1.13 | 1.15 | 1.11 | 2.72 | 10.52 | 8.44 | 7.83 | 16.44 | 14.93 | 15.91 | 11.24 | 12.77 | 12.88 | 117.86 | 8.42 | 35.71 |
| 14 | Citrate | 10.368 | 613586.25 | 18.12 | 28.71 | 17.10 | 16.78 | 12.47 | 11.24 | 3.82 | 3.48 | 0.02 | 0.05 | 0.05 | 0.03 | 0.19 | 0.22 | 112.28 | 8.02 | 87.29 |
| 15 | D-Cellobiose | 14.448 | 940908.06 | 10.24 | 14.00 | 19.43 | 12.93 | 9.42 | 0.10 | 0.27 | 2.07 | 4.77 | 6.62 | 5.54 | 7.85 | 7.15 | 7.58 | 107.97 | 7.71 | 28.73 |
| 16 | 1. O-Hexopyranosylhex-2-ulofuranose | 14.381 | 936257.75 | 0.21 | 0.21 | 0.88 | 0.17 | 0.61 | 5.51 | 5.60 | 24.18 | 21.14 | 8.73 | 9.35 | 9.49 | 9.58 | 10.38 | 106.03 | 7.57 | 57.26 |
| 17 | Melezitose | 19.377 | 1144837.25 | 1.64 | 1.76 | 5.41 | 5.92 | 3.73 | 14.22 | 9.42 | 16.24 | 11.65 | 11.71 | 10.06 | 4.23 | 5.26 | 3.72 | 104.96 | 7.50 | 21.93 |
| 18 | beta-D-Glucosamine | 11.005 | 664624.69 | 0.19 | 0.33 | 0.25 | 0.21 | 0.31 | 18.23 | 18.65 | 17.26 | 13.22 | 13.04 | 9.71 | 6.56 | 0.01 | 0.05 | 98.01 | 7.00 | 59.57 |
| 19 | alpha-D-Galactose | 10.773 | 646390.19 | 9.41 | 10.68 | 7.84 | 8.43 | 7.66 | 8.27 | 6.65 | 5.15 | 4.64 | 4.82 | 4.77 | 4.70 | 4.50 | 4.61 | 92.14 | 6.58 | 4.45 |
| 20 | 1-Kestose | 18.548 | 1119211.25 | 5.68 | 14.79 | 0.85 | 0.00 | 4.60 | 0.01 | 1.62 | 0.54 | 12.79 | 14.08 | 11.86 | 8.96 | 8.04 | 6.52 | 90.32 | 6.45 | 29.44 |
| 21 | 5-Deoxyribitol | 9.25 | 524269.34 | 2.06 | 5.11 | 2.41 | 3.97 | 0.05 | 10.44 | 10.40 | 8.21 | 7.24 | 7.55 | 7.57 | 7.11 | 6.85 | 10.03 | 89.00 | 6.36 | 10.43 |
| 22 | beta-Maltose | 14.452 | 941608.62 | 7.87 | 2.87 | 6.25 | 3.20 | 8.40 | 9.00 | 2.13 | 8.05 | 1.45 | 2.14 | 8.47 | 8.16 | 8.27 | 8.35 | 84.62 | 6.04 | 8.63 |
| 23 | beta-D-Fructose | 10.765 | 645830.56 | 10.10 | 12.69 | 8.33 | 9.32 | 8.81 | 7.04 | 6.60 | 3.24 | 3.41 | 3.45 | 3.88 | 0.00 | 2.10 | 0.52 | 79.50 | 5.68 | 14.81 |
| 24 | Turanose | 14.5 | 945119.19 | 4.70 | 5.18 | 3.79 | 5.93 | 2.86 | 3.14 | 3.69 | 3.45 | 5.46 | 6.59 | 7.48 | 7.52 | 8.74 | 8.91 | 77.43 | 5.53 | 4.24 |
| 25 | Sucrose | 14.176 | 920968.62 | 7.73 | 8.64 | 8.89 | 11.65 | 9.62 | 7.04 | 5.68 | 4.17 | 2.07 | 1.70 | 1.51 | 1.56 | 5.55 | 1.31 | 77.12 | 5.51 | 12.35 |
| 26 | L-(+)-Arabinose | 11.052 | 668748.81 | 0.75 | 0.38 | 1.01 | 1.08 | 0.90 | 4.85 | 6.68 | 7.42 | 6.71 | 8.23 | 8.28 | 7.77 | 9.83 | 11.15 | 75.03 | 5.36 | 14.41 |
| 27 | Salicin | 13.902 | 899837.94 | 0.28 | 1.12 | 0.48 | 0.78 | 0.61 | 3.28 | 2.95 | 2.79 | 5.83 | 7.11 | 10.33 | 12.62 | 11.59 | 9.60 | 69.38 | 4.96 | 20.25 |
| 28 | D-Mannitol | 10.991 | 663834.5 | 5.80 | 7.63 | 4.07 | 5.49 | 2.94 | 4.73 | 7.16 | 7.42 | 3.93 | 0.61 | 1.63 | 4.80 | 1.68 | 2.12 | 60.00 | 4.29 | 5.19 |
| 29 | Mellibiose | 14.91 | 973780.5 | 3.66 | 4.71 | 3.09 | 5.86 | 2.94 | 4.75 | 3.43 | 3.95 | 3.92 | 3.99 | 3.95 | 5.07 | 3.51 | 3.89 | 56.72 | 4.05 | 0.64 |
| 30 | Allose | 10.993 | 664342.19 | 5.59 | 7.35 | 4.30 | 5.01 | 4.80 | 3.60 | 4.30 | 0.72 | 2.62 | 2.98 | 2.98 | 0.62 | 5.38 | 5.85 | 56.09 | 4.01 | 3.63 |
| 31 | Sorbose | 10.99 | 664097.81 | 5.80 | 7.63 | 4.07 | 5.49 | 3.90 | 5.13 | 6.68 | 5.47 | 3.93 | 1.29 | 1.63 | 0.59 | 1.68 | 0.77 | 54.07 | 3.86 | 5.34 |
| 32 | D-Threonic acid | 8.855 | 492219.59 | 2.31 | 4.06 | 1.38 | 3.28 | 2.64 | 4.18 | 3.60 | 4.00 | 4.04 | 5.64 | 3.97 | 4.15 | 4.58 | 5.35 | 53.17 | 3.80 | 1.28 |
| 33 | D-Glucose | 11.183 | 679659.69 | 2.09 | 0.19 | 1.51 | 4.22 | 0.63 | 1.00 | 0.83 | 4.86 | 6.30 | 6.49 | 6.38 | 6.05 | 4.96 | 4.16 | 49.69 | 3.55 | 5.76 |
| 34 | Myo-Inositol | 11.302 | 689443.5 | 3.21 | 0.62 | 4.98 | 0.01 | 0.83 | 0.57 | 1.17 | 1.40 | 1.30 | 2.78 | 1.55 | 4.61 | 3.76 | 5.73 | 32.51 | 2.32 | 3.41 |
| 35 | D-Lyxose | 9.473 | 542114.56 | 0.32 | 0.44 | 1.13 | 1.80 | 0.43 | 3.62 | 4.45 | 4.53 | 3.92 | 5.12 | 4.77 | 5.04 | 4.99 | 5.98 | 46.53 | 3.32 | 4.16 |
| 36 | Erythrohexulose | 10.534 | 627864.94 | 0.47 | 0.72 | 1.45 | 0.87 | 2.81 | 8.59 | 11.93 | 14.70 | 0.82 | 0.06 | 0.65 | 0.03 | 1.29 | 1.08 | 45.47 | 3.25 | 23.07 |
| 37 | Hexuronic acid | 11.133 | 675984.75 | 0.85 | 0.78 | 2.33 | 1.02 | 4.08 | 0.29 | 6.36 | 5.55 | 4.41 | 3.88 | 3.95 | 6.19 | 0.43 | 0.54 | 40.67 | 2.90 | 5.12 |
| 38 | Polymaltose | 14.358 | 934441.94 | 2.09 | 2.78 | 1.82 | 3.54 | 2.90 | 1.76 | 1.58 | 1.66 | 2.19 | 2.69 | 3.33 | 3.82 | 4.66 | 4.81 | 39.64 | 2.83 | 1.16 |
| 39 | Lactobiose | 14.359 | 934546.38 | 0.71 | 2.78 | 1.89 | 3.58 | 2.90 | 1.76 | 1.41 | 1.51 | 2.63 | 3.19 | 2.93 | 4.39 | 4.66 | 4.81 | 39.15 | 2.80 | 1.59 |
| 40 | D-Galactonate | 11.28 | 687592.38 | 3.23 | 1.54 | 4.88 | 0.44 | 0.83 | 0.62 | 1.08 | 1.06 | 4.27 | 4.31 | 3.24 | 4.60 | 4.73 | 3.80 | 38.60 | 2.76 | 2.99 |
| 41 | alpha-d-Xylopyranose | 11.076 | 670893.38 | 2.25 | 1.28 | 2.38 | 4.86 | 2.21 | 0.92 | 1.82 | 0.26 | 3.05 | 3.45 | 3.23 | 2.73 | 4.15 | 4.13 | 36.73 | 2.62 | 1.70 |
| 42 | D-Arabinopyranose | 9.551 | 548084.81 | 3.08 | 1.72 | 3.16 | 4.59 | 3.38 | 1.47 | 3.25 | 3.35 | 1.46 | 1.20 | 1.48 | 1.58 | 2.70 | 3.87 | 36.28 | 2.59 | 1.18 |
| 43 | D-Xylonolactone | 9.482 | 543150.56 | 1.39 | 2.00 | 0.63 | 1.00 | 1.27 | 1.92 | 2.91 | 3.49 | 3.54 | 3.82 | 3.83 | 2.61 | 3.86 | 3.99 | 36.24 | 2.59 | 1.44 |
| 44 | Pentose | 9.487 | 543004.81 | 1.39 | 2.00 | 0.58 | 1.00 | 1.27 | 1.93 | 2.91 | 3.49 | 3.54 | 3.82 | 3.83 | 2.61 | 3.86 | 3.99 | 36.19 | 2.59 | 1.46 |
| 45 | 2,6-beta-delta-Fructan | 19.056 | 1135120.5 | 0.69 | 0.00 | 0.00 | 1.04 | 0.00 | 1.16 | 0.34 | 2.20 | 4.20 | 5.17 | 5.22 | 5.80 | 5.03 | 4.76 | 35.62 | 2.54 | 5.42 |
| 46 | 3,6-Anhydroglucose | 10.012 | 585153.19 | 3.41 | 4.79 | 2.62 | 4.08 | 3.40 | 3.36 | 3.32 | 2.65 | 2.14 | 1.07 | 1.64 | 0.02 | 1.09 | 0.86 | 34.44 | 2.46 | 1.89 |
| 47 | D-(+)-Cellobiose | 14.261 | 926683 | 1.35 | 1.20 | 1.30 | 1.88 | 2.75 | 0.07 | 0.78 | 0.91 | 2.50 | 3.74 | 3.80 | 4.54 | 4.44 | 5.07 | 34.32 | 2.45 | 2.62 |
| 48 | Lactulose | 14.266 | 927509.44 | 1.35 | 1.20 | 1.54 | 1.94 | 1.42 | 0.06 | 0.78 | 0.91 | 2.50 | 3.74 | 3.80 | 4.54 | 4.44 | 5.07 | 33.30 | 2.38 | 2.64 |
| 49 | D-Galactose | 10.721 | 642222.44 | 18.12 | 1.47 | 1.48 | 0.60 | 2.81 | 1.65 | 0.29 | 0.39 | 0.18 | 0.28 | 0.32 | 0.24 | 2.45 | 2.71 | 32.98 | 2.36 | 21.52 |
| 50 | 1. Gluconic acid, 2. delta-lactone | 10.811 | 649502.38 | 2.26 | 1.74 | 1.35 | 5.24 | 0.96 | 0.54 | 3.36 | 3.60 | 2.33 | 5.12 | 2.31 | 2.00 | 0.04 | 1.89 | 32.76 | 2.34 | 2.38 |
| 51 | 1. O-Methyl-D-   mannopyranosa | 10.421 | 617884.31 | 0.20 | 0.20 | 0.23 | 2.47 | 0.24 | 0.17 | 5.70 | 7.54 | 8.70 | 1.46 | 1.35 | 1.34 | 0.87 | 1.18 | 31.63 | 2.26 | 8.29 |
| 52 | Lactitol | 14.71 | 960055.5 | 1.32 | 1.51 | 0.59 | 1.50 | 0.96 | 0.91 | 0.95 | 0.87 | 2.10 | 2.55 | 3.41 | 4.55 | 4.38 | 4.78 | 30.40 | 2.17 | 2.26 |
| Acids and their derivatives | | | | | | | | | | | | | | | | | | | | |
| 53 | Phosphoric acid | 6.995 | 344011.38 | 22.58 | 31.95 | 29.35 | 29.31 | 26.70 | 45.30 | 34.70 | 30.93 | 38.01 | 42.32 | 26.15 | 39.80 | 32.26 | 29.57 | 458.95 | 32.78 | 42.43 |
| 54 | Malic acid | 8.447 | 459970.28 | 16.58 | 26.70 | 16.91 | 26.02 | 21.76 | 26.51 | 25.64 | 24.35 | 22.87 | 13.70 | 20.85 | 21.47 | 19.41 | 23.26 | 306.03 | 21.86 | 16.30 |
| 55 | Glycolic acid | 5.573 | 232280.52 | 6.78 | 5.67 | 7.35 | 7.34 | 8.61 | 19.60 | 25.56 | 26.83 | 28.62 | 31.74 | 30.19 | 33.72 | 34.69 | 37.79 | 304.49 | 21.75 | 146.27 |
| 56 | Lactic acid | 5.444 | 222124.02 | 7.04 | 8.84 | 11.21 | 24.09 | 8.34 | 14.14 | 15.51 | 16.24 | 16.26 | 18.33 | 17.43 | 18.70 | 18.73 | 20.32 | 215.19 | 15.37 | 24.46 |
| 57 | Hexadecanoic acid | 11.576 | 711487.06 | 12.68 | 17.16 | 11.09 | 15.34 | 13.69 | 15.42 | 15.87 | 15.89 | 15.57 | 15.40 | 15.19 | 14.28 | 17.05 | 16.00 | 210.63 | 15.05 | 2.70 |
| 58 | Octadecanoic acid | 12.477 | 785144.25 | 10.45 | 14.27 | 9.19 | 12.85 | 10.91 | 11.41 | 10.31 | 10.94 | 10.44 | 9.64 | 9.88 | 8.16 | 11.00 | 11.33 | 150.78 | 10.77 | 2.24 |
| 59 | D-Glyceric acid | 7.379 | 374229.88 | 4.33 | 2.00 | 1.66 | 1.68 | 4.96 | 9.72 | 9.42 | 9.91 | 10.26 | 11.43 | 11.68 | 11.98 | 14.11 | 15.56 | 118.71 | 8.48 | 21.93 |
| 60 | Maleic acid | 7.228 | 362465 | 3.31 | 2.24 | 2.92 | 3.54 | 2.79 | 2.75 | 8.40 | 8.35 | 5.48 | 7.46 | 9.62 | 9.21 | 8.02 | 8.16 | 82.26 | 5.88 | 7.96 |
| 61 | Oxalic acid | 5.957 | 262214 | 3.50 | 6.00 | 3.25 | 4.95 | 4.00 | 7.96 | 8.55 | 7.75 | 8.37 | 8.19 | 7.62 | 3.90 | 0.13 | 0.16 | 74.32 | 5.31 | 8.58 |
| 62 | Succinic acid | 7.298 | 367911.75 | 1.47 | 1.87 | 1.70 | 2.20 | 2.01 | 5.63 | 6.85 | 6.70 | 7.35 | 5.98 | 6.48 | 7.05 | 6.23 | 6.44 | 67.97 | 4.85 | 5.60 |
| 63 | Oxalacetic acid | 5.379 | 216583.58 | 0.51 | 0.68 | 1.64 | 3.89 | 6.01 | 6.42 | 5.43 | 6.61 | 4.78 | 5.71 | 4.42 | 5.21 | 5.82 | 10.12 | 67.23 | 4.80 | 6.49 |
| 64 | 2-Butene-1,4-dicarboxylic acid | 8.648 | 475807 | 7.73 | 13.83 | 8.40 | 13.89 | 10.71 | 0.93 | 0.94 | 1.50 | 0.17 | 3.56 | 2.19 | 1.98 | 0.80 | 0.56 | 67.20 | 4.80 | 25.62 |
| 65 | 4-Hydroxybutanoic acid | 6.728 | 322959.47 | 11.08 | 10.42 | 10.60 | 7.39 | 9.83 | 0.96 | 0.50 | 0.47 | 0.34 | 2.17 | 0.35 | 0.33 | 3.35 | 0.51 | 58.30 | 4.16 | 20.77 |
| 66 | Ethylmalonic acid | 7.201 | 359911.34 | 0.82 | 0.84 | 1.08 | 1.50 | 1.34 | 4.47 | 5.25 | 5.13 | 4.94 | 5.08 | 4.93 | 4.46 | 4.35 | 4.31 | 48.50 | 3.46 | 3.41 |
| 67 | 5-Hydroxymethyl-2-furancarboxylic acid | 8.858 | 492795.88 | 2.87 | 4.26 | 1.38 | 2.87 | 2.93 | 4.18 | 3.60 | 1.41 | 4.04 | 2.11 | 3.97 | 4.15 | 4.98 | 5.72 | 48.47 | 3.46 | 1.62 |
| 68 | Tartronic acid | 7.767 | 405325.09 | 4.70 | 4.07 | 4.44 | 5.65 | 4.20 | 3.29 | 3.14 | 0.00 | 2.75 | 3.12 | 3.00 | 3.09 | 3.08 | 3.11 | 47.65 | 3.40 | 1.66 |
| 69 | 2-Hydroxybutanoic acid | 8.067 | 429239.5 | 0.35 | 3.11 | 1.36 | 1.80 | 1.65 | 3.22 | 3.55 | 4.16 | 2.91 | 2.94 | 4.30 | 5.38 | 3.37 | 4.91 | 43.01 | 3.07 | 1.98 |
| 70 | Linoleate | 12.35 | 774773.75 | 0.96 | 2.03 | 0.74 | 1.42 | 2.87 | 3.20 | 2.67 | 3.54 | 3.65 | 2.56 | 3.30 | 3.40 | 4.42 | 3.04 | 37.80 | 2.70 | 1.14 |
| 71 | Phosphoenolpyruvate | 5.657 | 238525.5 | 1.56 | 1.82 | 1.78 | 2.09 | 1.25 | 1.28 | 1.43 | 1.79 | 1.75 | 1.87 | 1.53 | 1.78 | 6.56 | 6.99 | 33.48 | 2.39 | 3.51 |
| Amino acids and their derivatives | | | | | | | | | | | | | | | | | | | | |
| 72 | L-Pyroglutamic acid | 8.732 | 482187.94 | 38.39 | 46.30 | 39.34 | 47.70 | 41.95 | 41.12 | 39.27 | 40.33 | 31.57 | 31.55 | 26.42 | 14.56 | 35.28 | 40.29 | 514.11 | 36.72 | 73.37 |
| 73 | 5-Hydroxy-L-tryptophan | 14.108 | 915635.44 | 3.74 | 3.82 | 2.86 | 4.10 | 4.69 | 26.91 | 4.95 | 4.73 | 5.04 | 5.39 | 4.54 | 38.88 | 36.21 | 27.24 | 173.08 | 12.36 | 180.47 |
| 74 | gamma-Aminobutyric acid | 8.754 | 484401.06 | 27.94 | 33.44 | 16.85 | 24.90 | 26.43 | 0.16 | 0.02 | 0.13 | 0.03 | 0.08 | 0.08 | 0.03 | 0.09 | 0.09 | 130.26 | 9.30 | 176.10 |
| 75 | L-Proline | 7.224 | 361795.81 | 33.65 | 13.57 | 22.12 | 27.14 | 22.16 | 0.26 | 0.15 | 0.16 | 0.08 | 0.07 | 0.08 | 0.05 | 0.09 | 0.09 | 119.69 | 8.55 | 154.70 |
| 76 | L-Tyrosine | 11.037 | 667722.06 | 28.43 | 3.16 | 39.04 | 17.35 | 16.47 | 0.29 | 0.59 | 0.50 | 0.30 | 0.48 | 0.39 | 0.49 | 0.56 | 0.50 | 108.54 | 7.75 | 159.60 |
| 77 | L-Serine | 7.587 | 390457.97 | 0.05 | 18.47 | 27.27 | 33.41 | 23.93 | 0.17 | 0.03 | 0.01 | 0.01 | 0.10 | 0.12 | 0.14 | 0.18 | 0.17 | 104.05 | 7.43 | 153.87 |
| 78 | L-Isoleucine | 7.16 | 356962.56 | 29.68 | 20.92 | 30.34 | 0.45 | 19.59 | 0.28 | 0.21 | 0.22 | 0.06 | 0.05 | 0.05 | 0.11 | 0.04 | 0.05 | 102.04 | 7.29 | 144.56 |
| 79 | Glycine | 7.267 | 365452.56 | 15.54 | 13.65 | 16.24 | 21.20 | 15.86 | 0.09 | 0.06 | 0.08 | 0.06 | 0.06 | 0.06 | 0.07 | 0.09 | 0.11 | 83.17 | 5.94 | 69.12 |
| 80 | L-Leucine | 7.001 | 344367.44 | 30.45 | 6.33 | 20.78 | 9.48 | 15.39 | 0.00 | 0.00 | 0.01 | 0.05 | 0.06 | 0.05 | 0.07 | 0.05 | 0.04 | 82.75 | 5.91 | 95.09 |
| 81 | Carnitine | 6.72 | 322276.16 | 0.40 | 0.42 | 0.35 | 0.50 | 0.41 | 0.32 | 3.34 | 3.21 | 5.89 | 7.17 | 7.94 | 10.96 | 15.63 | 15.91 | 72.44 | 5.17 | 31.87 |
| 82 | Isoleucine | 6.372 | 294797.19 | 2.56 | 27.66 | 3.65 | 2.28 | 7.86 | 14.46 | 5.96 | 5.73 | 0.31 | 0.35 | 0.19 | 0.12 | 0.15 | 0.29 | 71.57 | 5.11 | 58.76 |
| 83 | L-Allothreonine | 7.765 | 405027.25 | 11.64 | 8.75 | 12.07 | 13.17 | 9.69 | 2.02 | 2.24 | 1.91 | 1.69 | 1.58 | 1.56 | 1.35 | 1.22 | 0.99 | 69.88 | 4.99 | 23.17 |
| 84 | DL-Alanine | 5.751 | 246069.06 | 0.12 | 9.98 | 20.28 | 11.89 | 22.03 | 0.59 | 0.43 | 0.41 | 0.47 | 0.53 | 0.56 | 0.60 | 0.01 | 0.01 | 67.92 | 4.85 | 62.30 |
| 85 | L-Pipecolate | 7.691 | 399180.75 | 14.84 | 2.22 | 21.08 | 13.25 | 9.08 | 0.36 | 0.12 | 0.11 | 0.09 | 0.04 | 0.09 | 0.05 | 0.00 | 0.01 | 61.35 | 4.38 | 50.70 |
| 86 | L-Phenylalanine | 9.354 | 532255.62 | 13.80 | 8.93 | 12.44 | 9.40 | 9.96 | 0.86 | 0.65 | 0.51 | 0.46 | 0.45 | 0.47 | 0.18 | 0.39 | 0.49 | 58.99 | 4.21 | 28.18 |
| 87 | L-Tryptophan | 12.364 | 776037.69 | 22.85 | 1.52 | 18.02 | 0.46 | 9.08 | 0.27 | 0.00 | 0.00 | 0.04 | 0.02 | 0.01 | 0.04 | 0.00 | 0.00 | 52.32 | 3.74 | 56.66 |
| 88 | L-Threonine | 7.173 | 357984.78 | 3.78 | 11.64 | 4.52 | 4.80 | 2.66 | 9.56 | 2.76 | 2.86 | 0.13 | 0.51 | 0.14 | 0.53 | 0.13 | 0.46 | 44.47 | 3.18 | 12.85 |
| 89 | L-Ornithine | 10.379 | 614696.19 | 10.38 | 2.19 | 14.89 | 3.76 | 8.20 | 0.06 | 0.21 | 0.23 | 0.34 | 0.31 | 0.30 | 0.27 | 0.30 | 0.30 | 41.73 | 2.98 | 22.46 |
| 90 | L-Citrulline | 10.42 | 617970.31 | 12.89 | 0.96 | 11.83 | 4.71 | 7.39 | 0.07 | 0.58 | 0.69 | 0.30 | 0.40 | 0.24 | 0.21 | 0.24 | 0.22 | 40.72 | 2.91 | 20.50 |
| 91 | DL-Leucine | 6.204 | 281817.06 | 1.55 | 12.95 | 1.59 | 0.69 | 6.30 | 7.57 | 3.93 | 4.04 | 0.25 | 0.06 | 0.06 | 0.14 | 0.14 | 0.16 | 39.44 | 2.82 | 14.70 |
| 92 | DL-Homoserine | 8.195 | 439290.97 | 3.67 | 2.37 | 9.07 | 8.35 | 12.67 | 0.18 | 0.10 | 0.10 | 0.03 | 0.07 | 0.04 | 0.06 | 0.03 | 0.05 | 36.80 | 2.63 | 18.08 |
| 93 | L-Methionine | 8.695 | 479508.5 | 7.66 | 5.72 | 10.05 | 5.68 | 5.88 | 0.12 | 0.11 | 0.10 | 0.06 | 0.05 | 0.06 | 0.02 | 0.05 | 0.05 | 35.59 | 2.54 | 12.98 |
| 94 | N-Acetylserotonin | 13.138 | 839007.25 | 0.47 | 0.77 | 0.75 | 1.33 | 1.73 | 3.92 | 4.22 | 3.57 | 3.85 | 4.40 | 3.85 | 3.71 | 0.92 | 0.83 | 34.33 | 2.45 | 2.48 |
| 95 | Glycyl-L-proline | 11.463 | 702390.19 | 8.57 | 7.87 | 5.01 | 4.19 | 8.13 | 0.01 | 0.01 | 0.01 | 0.03 | 0.03 | 0.03 | 0.04 | 0.03 | 0.02 | 33.98 | 2.43 | 12.44 |
| 96 | N-Acetylornithine | 11.463 | 702339.06 | 8.57 | 7.86 | 5.01 | 4.19 | 8.13 | 0.01 | 0.01 | 0.01 | 0.03 | 0.03 | 0.03 | 0.04 | 0.03 | 0.02 | 33.97 | 2.43 | 12.43 |
| 97 | L-Lysine | 10.935 | 659345.19 | 11.57 | 2.38 | 8.46 | 2.58 | 6.52 | 0.03 | 0.00 | 0.00 | 0.12 | 0.02 | 0.01 | 0.19 | 0.22 | 0.30 | 32.40 | 2.31 | 14.26 |
| 98 | L-Aspartate | 8.064 | 429175 | 4.93 | 11.20 | 3.17 | 3.12 | 3.82 | 2.06 | 0.41 | 0.50 | 0.09 | 0.09 | 0.07 | 0.06 | 0.01 | 0.04 | 29.57 | 2.11 | 9.73 |
| 99 | DL-Aspartic acid | 8.654 | 476290.22 | 10.10 | 2.76 | 6.19 | 5.61 | 3.16 | 0.10 | 0.16 | 0.20 | 0.14 | 0.20 | 0.05 | 0.07 | 0.06 | 0.02 | 28.81 | 2.06 | 10.02 |
| Nucleotides and theirderivatives | | | | | | | | | | | | | | | | | | | | |
| 100 | Cytosine arabinoside monophosphate | 13.377 | 858777.44 | 12.19 | 13.65 | 12.07 | 15.34 | 14.03 | 46.10 | 53.17 | 54.09 | 64.36 | 67.08 | 61.65 | 58.69 | 55.02 | 55.17 | 582.62 | 41.62 | 500.22 |
| 101 | 5-Methylcytosine | 8.807 | 488260.97 | 0.04 | 0.06 | 0.15 | 0.11 | 0.94 | 0.04 | 12.11 | 3.69 | 14.00 | 14.17 | 2.46 | 5.04 | 11.76 | 9.17 | 73.73 | 5.27 | 32.57 |
| 102 | 5-Methyluridine | 13.503 | 867551.62 | 0.12 | 0.57 | 2.55 | 3.29 | 4.83 | 4.85 | 3.49 | 3.29 | 3.17 | 3.01 | 2.73 | 2.73 | 0.85 | 1.02 | 36.50 | 2.61 | 2.17 |
| Esters and their derivatives | | | | | | | | | | | | | | | | | | | | |
| 103 | 3-Hydroxypropanoate | 6.066 | 271054.16 | 13.24 | 6.10 | 9.38 | 9.32 | 10.37 | 17.11 | 22.78 | 24.27 | 26.70 | 27.49 | 25.88 | 21.28 | 28.31 | 30.87 | 273.09 | 19.51 | 70.31 |
| 104 | Erythronic acid lactone | 8.12 | 433486.91 | 1.88 | 2.19 | 2.71 | 3.04 | 1.91 | 2.47 | 2.65 | 3.08 | 3.66 | 6.49 | 6.07 | 6.59 | 7.23 | 8.59 | 58.56 | 4.18 | 5.23 |
| 105 | D-Mannonate | 11.294 | 688651.19 | 3.23 | 0.67 | 4.88 | 0.44 | 0.83 | 0.57 | 1.12 | 1.47 | 2.24 | 4.31 | 3.86 | 4.61 | 4.58 | 9.25 | 42.05 | 3.00 | 6.10 |
| 106 | 1. Hydroxy-3   -methylglutarate | 9.157 | 516666.41 | 1.79 | 1.71 | 1.99 | 2.55 | 1.57 | 2.30 | 4.66 | 1.19 | 3.55 | 4.19 | 3.34 | 2.36 | 2.07 | 1.64 | 34.91 | 2.49 | 1.10 |
| Flavonoid | | | | | | | | | | | | | | | | | | | | |
| 107 | Neohesperidin | 13.564 | 873365.69 | 2.56 | 2.23 | 3.36 | 3.99 | 3.82 | 12.21 | 8.04 | 7.06 | 5.86 | 6.25 | 6.70 | 6.19 | 5.14 | 5.53 | 78.97 | 5.64 | 6.56 |
| Vitamins | | | | | | | | | | | | | | | | | | | | |
| 108 | Nicotinic acid | 7.254 | 364359.28 | 3.32 | 4.72 | 2.66 | 3.65 | 2.91 | 8.27 | 3.24 | 3.57 | 2.49 | 3.14 | 2.79 | 2.78 | 3.23 | 5.29 | 52.05 | 3.72 | 2.33 |
| 109 | Pantothenic acid | 11.272 | 686834.94 | 1.57 | 0.81 | 2.30 | 0.42 | 0.40 | 0.43 | 3.44 | 3.38 | 3.63 | 4.16 | 3.82 | 3.49 | 2.32 | 1.50 | 31.67 | 2.26 | 1.96 |
| 110 | Ascorbate | 11.041 | 668243.88 | 0.87 | 8.75 | 0.72 | 0.24 | 0.52 | 3.81 | 1.90 | 0.18 | 0.03 | 1.92 | 1.96 | 2.25 | 2.43 | 2.37 | 27.95 | 2.00 | 4.97 |
| Miscellaneous | | | | | | | | | | | | | | | | | | | | |
| 111 | 2-Hydroxypyridine | 5.31 | 211191.23 | 21.18 | 25.65 | 19.12 | 9.88 | 20.40 | 16.71 | 17.13 | 17.26 | 18.61 | 22.67 | 21.39 | 23.86 | 6.64 | 26.72 | 267.22 | 19.09 | 30.85 |
| 112 | 2-(3,4-Dihydroxyoxolan-2-yl)-2-hydroxyacetaldehyde | 10.005 | 584461.12 | 0.81 | 1.11 | 0.03 | 1.11 | 2.70 | 10.52 | 14.62 | 12.22 | 16.44 | 14.93 | 15.91 | 15.11 | 14.28 | 12.88 | 132.67 | 9.48 | 43.93 |
| 113 | 3-Hydroxypyridine | 6.097 | 273445.56 | 1.30 | 1.70 | 1.32 | 1.57 | 1.25 | 4.60 | 6.51 | 7.00 | 11.83 | 13.79 | 11.68 | 9.10 | 22.77 | 21.18 | 115.59 | 8.26 | 52.69 |
| 114 | 2-Pyrrolidinone | 6.15 | 277417.94 | 9.96 | 10.42 | 4.61 | 4.73 | 7.66 | 3.04 | 3.45 | 4.40 | 1.97 | 1.26 | 1.29 | 0.48 | 0.50 | 0.63 | 54.39 | 3.89 | 11.33 |
| 115 | (2R,3R,4S,5S,6R)-2-[(2S,3S,4S,5R)-2-[[(2S,3S,4S,5R)-2-Ethyl-3,4-dihydroxy-5-(hydroxymethyl)oxolan-2-yl]peroxymethyl]-3,4-dihydroxy-5-(hydroxymethyl)oxolan-2-yl]oxy-6-(hydroxymethyl)oxane-3,4,5-triol | 19.927 | 1162007.88 | 2.08 | 1.23 | 2.61 | 1.27 | 0.56 | 11.08 | 9.77 | 5.84 | 0.78 | 1.21 | 5.82 | 0.23 | 0.18 | 0.13 | 42.81 | 3.06 | 13.25 |
| 116 | Urea | 6.828 | 330784.88 | 7.39 | 6.10 | 8.64 | 4.44 | 3.52 | 1.66 | 0.28 | 0.35 | 0.10 | 0.07 | 0.05 | 0.25 | 0.31 | 0.78 | 33.93 | 2.42 | 9.23 |
